# All-Biobased Hydrovoltaic-Photovoltaic Electricity Generators for All-Weather Energy Harvesting

**DOI:** 10.34133/2022/9873203

**Published:** 2022-08-20

**Authors:** Guoping Ren, Qichang Hu, Jie Ye, Andong Hu, Jian Lü, Shungui Zhou

**Affiliations:** ^1^College of Resources and Environment, Fujian Agriculture and Forestry University, Fuzhou, China; ^2^College of Mechanical and Electrical Engineering, Fujian Agriculture and Forestry University, Fuzhou, China

## Abstract

Hygroelectricity generators (HEGs) utilize the latent heat stored in environmental moisture for electricity generation, but nevertheless are showing relatively low power densities due to their weak energy harvesting capacities. Inspired by epiphytes that absorb ambient moisture and concurrently capture sunlight for dynamic photosynthesis, we propose herein a scenario of all-biobased hydrovoltaic-photovoltaic electricity generators (HPEGs) that integrate photosystem II (PSII) with *Geobacter sulfurreducens* (*G.s*) for simultaneous energy harvesting from both moisture and sunlight. This proof of concept illustrates that the all-biobased HPEG generates steady hygroelectricity induced by moisture absorption and meanwhile creates a photovoltaic electric field which further strengthens electricity generation under sunlight. Under environmental conditions, the synergic hydrovoltaic-photovoltaic effect in HPEGs has resulted in a continuous output power with a high density of 1.24 W/m^2^, surpassing all HEGs reported hitherto. This work thus provides a feasible strategy for boosting electricity generation via simultaneous energy harvesting from ambient moisture and sunlight.

## 1. Introduction

Spontaneous power generation that converts natural energy into electric energy (such as thermoelectricity, photoelectricity, and triboelectricity) has been considered as a promising technology to supplement the vast demand for electricity [1–5]. Although the performance of these power generation technologies has been greatly improved, the instability (such as diurnal variation of solar energy and certain temperature difference) of energy sources limits their continuous and sustainable electricity generation [1, 6]. As known, water media on Earth absorbs about 35% of the solar radiation energy reaching the earth surface (equivalent to 6.0 × 10^16^ W) [4], which would be sufficient for an annual energy requirement for all mankind (ca. 1.8 × 10^13^ W) as long as one thousandth of the energy stored in water is harnessed. Recent studies have shown that interactions between low-dimensional carbon materials and water are capable of initiating power generation, the so-called hydrovoltaic effect, including droplet electricity generation, evaporation electricity generation, and hygroelectricity generation [7–10]. In particular, hygroelectricity generators (HEGs) have recently attracted enormous research attention due to their applicability in utilizing the unlimited, pollution-free, and ubiquitous water vapor in the environment for power generation. The HEG was first reported to successfully generate a transient power density of 0.42 *μ*W/cm^2^ using a graphene oxide (GO) film, and the power density was further improved to 27 *μ*W/cm^2^ using asymmetrical GO [11, 12]. In addition to GO, metal-based nanomaterials were also studied in HEGs to harvest energy from ambient humidity [13]. However, such electricity generation with moisture can merely subsist for tens of seconds; nevertheless, the sustained output power of HEG systems has been achieved through sophisticated technical improvements [9, 14]. In a previous study, our team reported that microbial films constructed from whole-cell *Geobacter sulfurreducens* (*G.s*) behaved as reliable HEGs with sustained output power of a few voltages [15–17]. However, the structural features and component characteristics of whole-cell HEGs largely restricted their availability of harvesting other sources of energy in the environment (for example, the solar energy, as the one largest energy source in nature) [18, 19], except for water vapor, to further enhance the efficiency of power generation. From this point of view, it remains challenging to realize sustainable and stable electricity generators that simultaneously harvest solar energy and latent heat.

In nature, the evolution of abundant water absorption structures in leaves of epiphytes has been a sequel of their underdeveloped roots with unsatisfactory water taking [20, 21]. In addition, the photosynthetic system in leaves of these plants is able to capture and convert solar energy to maintain the growth and reproduction of plants. Therefore, the combination of photosynthetic systems and water/moisture absorption structures might be a feasible strategy to develop more advanced electricity generation systems, in which hybrids of well-united hydrovoltaic and photovoltaic counterparts synergically drive electricity generation to create next-generation electricity generators. However, the photosystem is composed of two light-capturing centers (photosystem I (PSI) and II (PSII)) in a Z scheme [22], and the complete electron transportation chain between PSI and PSII produces oxygen (O_2_) and reduces intracellular redox substances (such as NADP^+^/NADPH). Previous studies have demonstrated that Z-scheme photosensitive solar cells were successfully assembled from extracted PSII particles and further combination with photosensitive entities [22, 23]. Therefore, a scientific hypothesis is proposed to build novel all-biobased hydrovoltaic-photovoltaic electricity generators (HPEGs) through the hybridization of PSII with readily photosensitive counterparts possessing built-in HEG performances.

*G.s* contains abundant outer membrane c-type cytochromes (OM c-Cyts), which are known as photosensitizers and are able to be excited by light to produce photoelectrons for enhanced bioelectricity generation [24]. Moreover, *G.s* cells have shown HEG capacities featured by their distinctive biological processes of electron transport. Therefore, the combinational use of *G.s* and PSII is rational for creating biomimetic all-biobased systems that are potentially capable of utilizing dual energy resources in the environment, namely, moisture and sunlight, for viable electricity generation. In this work, all-biobased HPEGs were designed and assembled from whole-cell *G.s* and natural PSII by means of a facile film forming process. In practice, the all-biobased HPEGs achieved a sustainable and stable electricity generation due to an efficient separation of photoexcited electrons (e^−^) and holes (h^+^) at the OM c-Cyt-PSII interface, in which key limitations of overlapping light capture bands in the natural photochemical system were addressed. Moreover, the as-prepared all-biobased HPEGs were able to capture both the latent heat stored in environmental moisture and the solar energy for spontaneous power generation. Benefitting from the synergic hydrovoltaic-photovoltaic effect, the total output power (1.23 *μ*W) of an HPEG was ~1.73-fold higher than that of a sole hydrovoltaic (0.45 *μ*W) electricity generation. Furthermore, the all-biobased HPEG exhibited a continuous output power density of 1.24 W/m^2^, which surpassed all hygroelectricity generators reported hitherto.

## 2. Results

### 2.1. Characterization of *G.s*-PSII HPEGs

**Figure 1:**
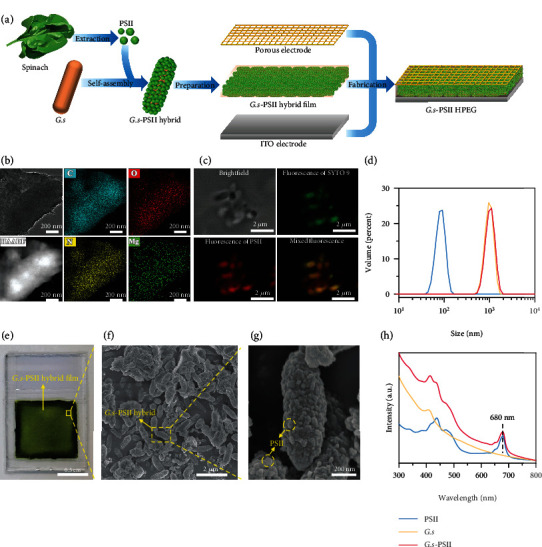
Fabrication and characterization of *G.s*-PSII HPEG. (a) The fabrication process of *G.s*-PSII hybrid film and HPEG, where the *G.s*-PSII hybrid film is sandwiched between the ITO glass and porous electrode to form *G.s*-PSII HPEG. (b) TEM and mapping images of *G.s*-PSII hybrid. (c) Brightfield and fluorescence images of the SYTO 9 labeled *G.s* cells and PSII particles. (d) Size distributions of PSII particles, *G.s* cells, and *G.s*-PSII hybrids. (e) Photograph of a *G.s*-PSII hybrid film. (f) SEM image of a *G.s*-PSII hybrid film. (g) SEM image of a *G.s*-PSII hybrid. (h) UV-vis absorption spectra.

An all-biobased *G.s*-PSII hybrid film was constructed by the hybridization of PSII particles and whole-cell *G.s* (Figure 1(a)). Briefly, PSII with particle sizes ranging from ca. 20 to 40 nm [25], as indicated from a transmission electron microscopy (TEM) image (Figure S1), was first extracted from the leaves of spinach by previously reported methods [26, 27]. Then, PSII particles were added to a suspension of *G.s* (Figure S2) in order to prepare *G.s*-PSII hybrids. The hybrids were centrifuged, resuspended, and added to the conductive side of an indium tin oxide (ITO) glass slide to form a *G.s*-PSII hybrid film (film area = 1.0 × 1.0 cm^2^) upon drying at 25°C for 24 h. Finally, a titanium mesh (1.0 × 1.0 cm^2^) was fixed on top of a *G.s*-PSII hybrid film electrode to fabricate the all-biobased HPEG, in a way that the *G.s*-PSII hybrid film was sandwiched between a titanium mesh and ITO glass slide as shown in Figure 1(a).

Surface compositions and characteristics of *G.s*-PSII hybrids were studied by high angle annular dark field (HAADF) in scanning TEM and energy-dispersive X-ray spectroscopic (EDS) mapping images (Figure 1(b)), indicating the hybrids were mainly composed of C, O, N, and Mg elements, which was assigned to Mg-containing chlorophyll monomers in PSII particles [25, 28]. Additionally, autofluorescence of chlorophylls was observed for *G.s*-PSII hybrids [29], which were in alignment with the shapes of *G.s* cells obtained by confocal laser-scanning microscope (CLSM) (Figure 1(c)). Moreover, size distribution analysis revealed that nearly no isolated PSII particles existed in the suspension (Figure 1(d)), suggesting the formation of a uniform system of *G.s*-PSII hybrids, which was consistent with the fluorescence and EDS mapping results.

As shown in Figure 1(e), the *G.s*-PSII hybrid film showed a dark green color due to the hybridization of PSII particles (bright green) and *G.s* cells (light red) (Figure S3). The presence of *G.s*-PSII hybrids in an all-biobased film was also demonstrated by scanning electron microscopy (SEM) images in which homogeneous rod-shaped *G.s* (Figure 1(f)) and decorated PSII nanoparticles (Figure 1(g)) were clearly identified. Surprisingly, the *G.s*-PSII hybrid film exhibited improved hydrophilicity in comparison with the *G.s* film (Figure S4), largely due to the modification of PSII, which is beneficial to water absorption [15]. Meanwhile, the UV-vis spectrum of *G.s*-PSII displayed an extended range of light absorption which was conductive to the enhanced utilization of sunlight (Figure 1(h)). These results implied that PSII particles were well integrated with whole-cell *G.s* into a *G.s*-PSII hybrid film with readily available biomimetic structure for moisture and light absorptions.

### 2.2. Performance of Electricity Generation for *G.s*-PSII HPEGs

**Figure 2:**
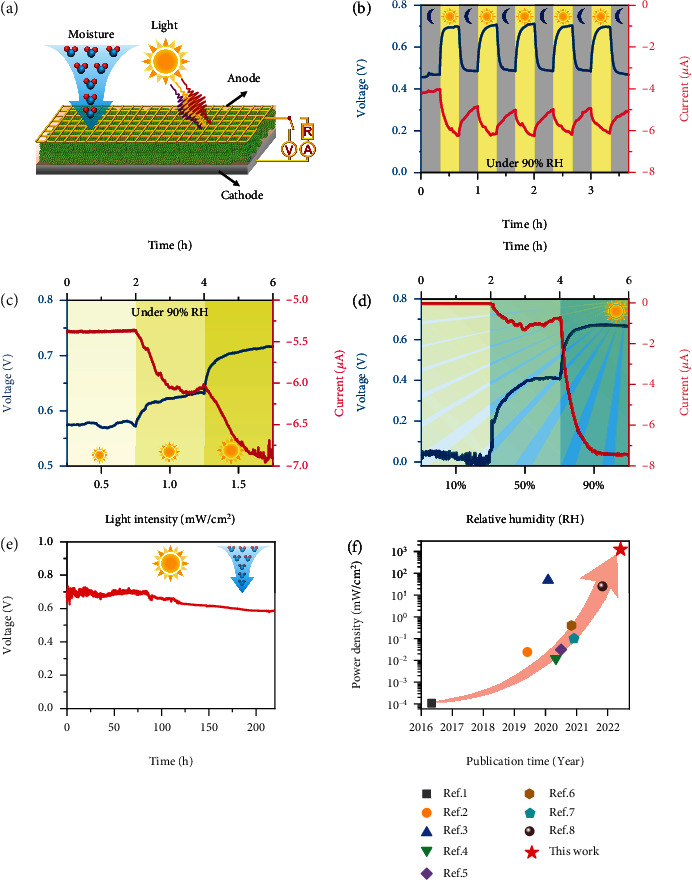
Electric output performance of the *G.s*-PSII HPEG. (a) Schematic diagram of the HPEG to generate electricity. (b) Electric output of the HPEG responds to intermittent light-darkness at 90%RH, where the grey and yellow backgrounds are in darkness and light, respectively (25 ± 2°C). (c) Continuous voltage and current measurements under different light intensities (0.5, 1.0, and 1.5 mW/cm^2^) at 90%RH (25 ± 2°C). (d) Continuous voltage and current measurements at different RHs (10%, 50%, and 90%RH) under a light intensity of 1.5 mW/cm^2^ (25 ± 2°C). (e) Long-time test of voltage under light at 90%RH and 25 ± 2°C. (f) A comparison of the power density of this current device with those of the sustainable hygroelectricity generators in the literature (Table S1).

As illustrated in Figure 2(a), HPEGs could potentially generate electricity by using the ambient humidity and light. To accurately measure the capacity of power generation, a HPEG was placed in an electricity testing system equipped with humidity- and light-controlling modules. The HPEG was able to generate an open-circuit voltage (*V*_oc_) of ~0.45 V and short-circuit current (*I*_sc_) of ~4.2 *μ*A at ca. 90% relative humidity (RH) in the absence of light (Figure 2(b)). Upon exposure to a full-spectrum light (1.5 mW/cm^2^) at 25 ± 2°C, *V*_oc_ and *I*_sc_ of the HPEG were significantly increased by 55% (to ~0.70 V) and 60% (to ~6.4 *μ*A), respectively. Alternatively, low electricity generation with *V*_oc_ of ~0.03 V and *I*_sc_ of ~1.0 nA was observed under light in the lack of moisture (~10%RH) (Figure S5). To further study the effect of light illumination, various light intensities were applied to an HPEG in continuous electricity generation tests, in which both *V*_oc_ and *I*_sc_ displayed progressive increases with increasing light intensities (Figure 2(c)). Specifically, *V*_oc_ increased from ~0.58 V (with a light intensity of 0.5 mW/cm^2^) to ~0.72 V (with a light intensity of 1.5 mW/cm^2^), and correspondingly, *I*_sc_ increased from ~5.4 *μ*A to ~6.9 *μ*A. Of note, application of a light with intensity up to 1.5 mW/cm^2^ only increased the temperature by ~1°C, which hardly affected the activity of *G.s*-PSII hybrid film (Figure S6).

Meanwhile, the ambient moisture was able to significantly influence the hydrovoltaic electricity generation of HPEGs. As shown in Figure 2(d), nearly no electricity was observed at low humidity (10%RH) with a light intensity of 1.5 mW/cm^2^, whereas *V*_oc_ and *I*_sc_ greatly increased to ~0.40 V and~1.2 *μ*A at 50%RH and to ~0.70 V and~7.2 *μ*A at 90%RH, respectively. Furthermore, no electricity generation was observed in the absence of moisture and light (Figure S7(a)). Concurrently, the light illumination would improve the electricity (Figure S7(b)). In addition, HPEGs fabricated with various electrodes (glassy carbon, Au, and Cu) resulted in comparable *V*_oc_ and *I*_sc_, suggesting the electricity generation was originated from hydrovoltaic and photovoltaic effects rather than redox reactions of electrodes (Figure S8). In comparison to conventional hydrovoltaic electricity generation systems [9, 11, 30], the all-biobased HPEGs were able to simultaneously capture energy from moisture and light to enhance output power.

The effect of PSII content, size, and thickness of *G.s*-PSII hybrid films on the performance of HPEGs was also studied. Considering the excellent light absorption ability of PSII, a HPEG with 20 wt% of PSII was sufficient to generate optimal voltage and current (Figure S9(a)). Likewise, a film thickness of ~30 *μ*m (equal to a content of 1.5 mg/cm^2^*G.s*-PSII) achieved comparable *V*_oc_ and *I*_sc_ (Figure S9(b)) due to efficient ion dissociation and diffusion within a hybrid film [10]. Upon connecting with an optimized resistance of 64 k*Ω* (equal to an internal resistance of HPEG), the optimal HPEG was able to deliver a power density of 14.13 mW/m^2^ (Figure S10). Furthermore, changes in film sizes significantly affected the output of currents rather than voltages (Figure S11). Accordingly, an output power density of 173 mW/m^2^ was generated at 90%RH with a light intensity of 1.5 mW/cm^2^. Surprisingly, the electricity output decreased by only ~8.0% after 220 h (Figure 2(e)), indicating a steady performance of the *G.s*-PSII HPEG. The continuous electricity generation was mainly due to the water adsorption-desorption process [9, 16]. Abundant pores created the interspace of films that were associated with the external environment. In such circumstance, water molecules were trapped into the pore space due to evaporation and then escaped to the atmosphere driven by the difference of water vapor pressure between the internal pore and the external environment. Accordingly, the aqueous water molecules in the films were released into the external environment by the interspace, which induced a continuous water adsorption. Furthermore, the output power density could be improved to 1.24 W/m^2^ under a light intensity of 100 mW/cm^2^ (1.5 AM) at a humidity of 90%RH (Figure S12), which was among the highest numbers reported in the literature (Figure 2(f) and Table S1). Overall, the all-biobased *G.s*-PSII HPEGs could sustainably generate electricity which was synergistically induced by both moisture and light.

### 2.3. All-Weather Applications

**Figure 3:**
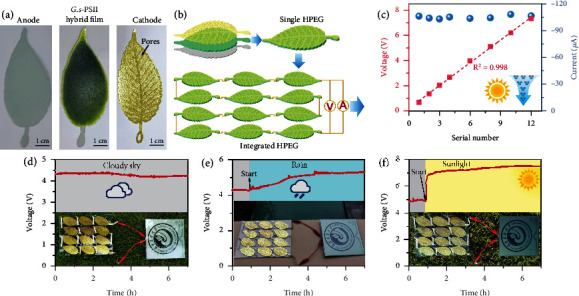
Demonstration of the *G.s*-PSII HPEG for all-weather power output. (a) Photographs of leaf-shaped ITO electrode, *G.s*-PSII hybrid film, and porous electrode. (b) Fabrication process of leaf-shaped HPEG and the integrated HPEG by connecting 12 leaf-shaped HPEG in series. (c) Voltage and current of the integrated HPEG varying with the number of series. (d) Output voltage of the integrated HPEG on cloudy sky, which can directly power the electronic ink screen (15 × 15 cm^2^). (e) Output voltage of the integrated HPEG on rainy day, which can power the electronic ink screen. (f) Output voltage of the integrated HPEG on sunny day, which can power the electronic ink screen. Photo credit: Guoping Ren, Fujian Agriculture and Forestry University.

The scalable integration of HPEGs is vital for their application in an ambient environment. Herein, leaf-shaped HPEGs were fabricated (Figure 3(a)), where each *G.s*-PSII hybrid film was sandwiched between a polyethylene terephthalate- (PET-) ITO electrode and a porous leaf-shaped electrode (Figure 3(b)). These leaf-shaped HPEGs were connected in series to form an integrated HPEG, whose output voltage increased linearly with the number of *G.s*-PSII HPEG modules under light illumination at ambient humidity (Figure 3(c)). Meanwhile, the output current of an integrated HPEG was basically maintained at a high level of ~105 *μ*A. Therefore, the integrated device displayed high performance of output power in an ambient environment, which was further investigated under realistic weather conditions. In general, the integrated HPEG generated a voltage of ~4.2 V on a cloudy day, which was sufficient to directly power an electronic ink screen (Figure 3(d), Figure S13, and Movie S1). As expected, the output voltage would increase to ~5.0 V on a rainy day and was further boosted to a higher value of ~7.5 V on a sunny day (Figures 3(e) and 3(f); Movies S2 and S3). These results demonstrated that integrated *G.s*-PSII HPEGs could sustain a stable output power in actual weather conditions. Therefore, the all-biobased *G.s*-PSII HPEG was applicable to all-weather electricity generation by harvesting sustainable and green energy from moisture and sunlight in the environment.

### 2.4. Mechanisms of Electricity Generation for *G.s*-PSII HPEGs

**Figure 4:**
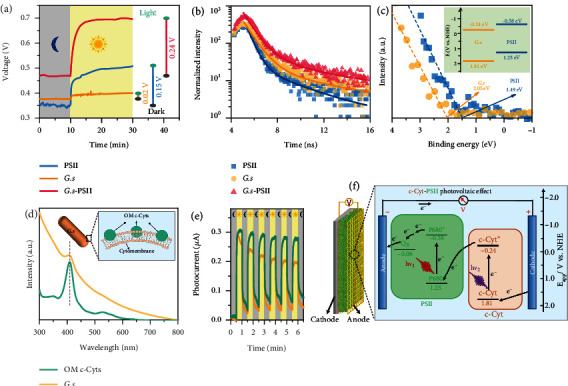
Electric output enhancement of the HPEG by photovoltaic effect of *G.s*-PSII hybrid. (a) Effect of light on electric output of the HPEGs based on the PSII film, *G.s* film, and *G.s*-PSII hybrid film at 90%RH, where the grey and yellow backgrounds are in darkness and light, respectively. (b) PL delay spectra of different films by time-correlated single photon counting. (c) VB XPS spectra of the PSII film and *G.s* film, where the inset is energy band diagram. (d) UV-vis absorption spectra of the *G.s* and the extracted OM c-Cyts, where the OM c-Cyts is on the cytomembrane of *G.s* in the inset. (e) The photoelectric *I* − *t* curves of the *G.s* and the OM c-Cyts under a light-darkness test (30/30 s). (f) Photoelectron transfer pathway between *G.s* and PSII and the relevant potentials in the *G.s*-PSII HPEG. c-Cyt: c-type cytochrome; P680: primary electron donor chlorophyll; Q_B_: plastoquinone B; “∗” indicates an excited state.

In the system of all-biobased *G.s*-PSII HPEGs, the high-performance electricity generation is mainly attributable to the enhanced capacity of light absorption and moisture utilization. However, the underlying mechanism remained unclear and thus was investigated in detail. In terms of light absorption and conversion, single-component PSII or *G.s* was fabricated into HPEGs for electricity generation by following the same fabrication process of *G.s*-PSII HPEGs. Of note, the PSII-based HPEG displayed an output voltage of 0.35 V (in dark) and 0.50 V (under light) at 90%RH (Figure 4(a)). Similarly, the *G.s*-based HPEG showed an output voltage of 0.38 V (in dark) and 0.40 V (under light) at 90%RH. By contrast, the *G.s*-PSII HPEGs generated slightly higher output voltages of 0.46 V (in dark) and 0.70 V (under light) at 90%RH. The overall increase in output voltage induced by all-biobased *G.s*-PSII HPEGs (~0.24 V) was considerably higher than that of PSII (~0.15 V) and *G.s* (~0.02 V). We inferred that the hybridization of PSII and *G.s* into all-biobased *G.s*-PSII HPEGs created unique local structures and interfacial boundaries that, on the one hand, improved light absorption capability and, on the other hand, facilitated to prompt separation of photoexcited electrons and holes upon light illumination.

The possible photo-induced electron transfer efficiency and recombination rate of carriers (electron-hole pairs) were evaluated by photoluminescence (PL) [31]. The PL emission spectra of PSII, *G.s*, and *G.s*-PSII hybrid all displayed emission bands between 700 and 760 nm (Figure S14), which were attributed to the band gap emission [31]. The weaker PL peak of *G.s*-PSII hybrid indicated a lower recombination rate of photocarriers [32]. To further verify the lifetime of photocarriers, PL delay spectra were measured and analyzed [31], by which various delay times were obtained in the order of *G*.*s*‐PSII > *G*.*s* > PSII (Figure 4(b) and Table S2). These results implied that the hybridization between PSII and *G.s* was able to sustain the excited state of carriers for a considerably longer time and the stayed carriers were more likely transfer to a cathode/anode for building an external electric field [31]. Furthermore, the valence band (VB) energy (*E*_VB_) of PSII and *G.s* was determined to be 1.49 eV and 2.05 eV, respectively (Figure 4(c)). Combined with the bandgaps (*E*_g_) of PSII and *G.s* [33, 34] (Figure S15), the conduction band (CB) energy (*E*_CB_) was calculated based on the equation of *E*_CB_ = *E*_VB_ − *E*_g_ [35] to be -0.34 and 0.0 eV, respectively. The band structures of *G.s* and PSII were estimated based on the equation of *E*_NHE_ = *Φ* − 4.44 + *E*_VB_ (*E*_NHE_: potential of normal hydrogen electrode; *Φ* (4.2 eV): electron work function of the XPS analyzer) [36]. The possible alignment of *E*_CB_ and *E*_VB_ of PSII and *G.s* was elucidated as shown in Figure 4(c) (inset), which was conductive for the transfer of carriers between PSII and *G.s* [37].

To further characterize the transfer of photocarriers in the system of *G.s*-PSII hybrids, the photosensitivity of *G.s* was first verified. Both *G.s* and OM c-Cyt extracted from *G.s* showed similar absorption peaks centered at 410 nm (Figure 4(d)). Moreover, the band structures of *G.s* and OM c-Cyt were comparable (Figure S16) and both generated reliable photocurrents under 0.4 V vs. the saturated calomel electrode (SCE) (Figure 4(e)). It was therefore concluded that OM c-Cyt was a vital photosensitizer in a *G.s*-PSII HPEG, which was confirmed by OM c-Cyt removal and addition experiments (Figure S17 and S18). *G.s* treated with proteinase K displayed significantly decreased output power, whereas the addition of OM c-Cyts to *G.s* could significantly increase the output power.

Based on the above results, a photovoltaic mechanism of *G.s*-PSII HPEGs is first proposed as follows (Figure 4(f)). Under light illumination, photoexcited electrons at the CB of OM c-Cyt are transferred to the VB of primary electron donor chlorophyll (P680) for quenching holes that are generated at the excited energy level P680 (P680∗). Photoelectrons at P680∗ are received by electron acceptor plastoquinone B (Q_B_) and further transferred to the anode [22]. Afterwards, the electrons transported from the anode to cathode by external circuit quench holes at the VB of OM c-Cyt. In such a way, the photoelectron transfer induces a photovoltaic electric field (*E*_P_), which sustainably generates electricity by harvesting energy from light. In addition, no hydrogen was detected during electricity generation, indicating that OM c-Cyt hardly reacted with protons.

**Figure 5:**
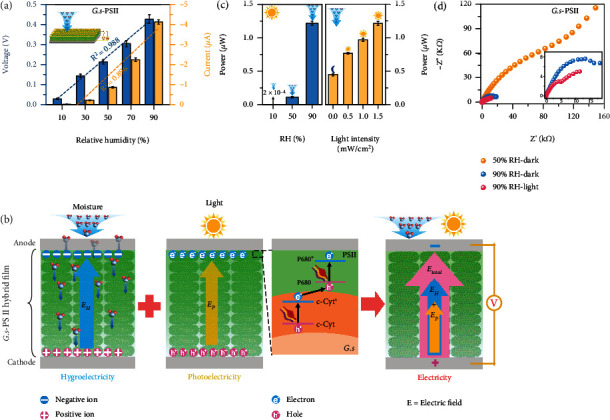
Coupled hygroelectric-photoelectric mechanism for electricity generation. (a) The effect of RHs on output voltage and current of the *G.s*-PSII HPEG under darkness. (b) Schematic diagram of the electric field formation by hydrovoltaic and photovoltaic effects in the HPEG (*E*_H_: hydrovoltaic electric field; *E*_P_: photovoltaic electric field; *E*_total_: total electric field). (c) Effect of different RHs and light intensities on output power of the HPEG. (d) EIS Nyquist plots of the HPEG. Inset: EIS Nyquist plots of the HPEG under dark and light at 90%RH.

Beyond *E*_P_, a hydrovoltaic electric field (*E*_H_) also exists in the all-biobased HPEG (Figure S19). According to Figure S20 and the literature [9, 16], a moisture gradient existed in the film. Upon exposure to the ambient moisture, cations (such as H^+^) may dissociate from -COOH moieties in the *G.s*-PSII hybrid film (Figure S21). Mobile cations diffuse into the interior of the film with water movement, which was shown to induce concentration gradients across cations [10, 38] and the formation of a diffusion current and *E*_H_. Notably, the porous structure of a hybrid film was conductive to the adsorption of water molecules into the pores through interfacial interactions [16, 39] and the diffusion outside the film through evaporation. Such a dynamic water adsorption-desorption process driven by the environmental energy effectively maintains the concentration gradients of charges (Figure S22). Accordingly, the *G.s*-PSII HPEG generates a sustainable hygroelectricity via the spontaneous water adsorption-desorption and the diffusion of charged ions. The mechanism is further verified by a long-term hygroelectricity generation test (Figure 2(f)), in which the output voltage and current increase with increasing relative humidity and coincided with results of pure *G.s* and PSII (Figure 5(a) and S23). This is attributable to the high ion conductivity and zeta potential of *G.s*-PSII hybrids (Figure S24), which reflects strong ion diffusion and dissociation, respectively, in coordination with the prosed mechanism for hygroelectricity generation.

These results demonstrate that a total electric field (*E*_total_) is generated by coupling hydrovoltaic and photovoltaic effects as shown in Figure 5(b). Upon exposure to moisture, *G.s*-PSII HPEG induces the formation of ion concentration difference and creates *E*_H_. Under light illumination, photoexcited electrons formed at c-Cyt∗ in *G.s* of *G.s*-PSII HPEG are transferred to the P680 of PSII and combine with holes, by which an electric field (*E*_P_) forms between the photoexcited electrons of PSII and holes of c-Cyt.

## 3. Discussion

Inspired by the biological structure of epiphytes leaves that concurrently capture ambient moisture and sunlight for photosynthesis [21], we developed an all-biobased HPEG based on *G.s*-PSII hybrid film in this study. In comparison to traditional HEGs, the *G.s*-PSII HPEGs exhibited much enhanced output power due to the efficient utilization of environmental solar energy. The optimum output power density reached to 1.24 W/m^2^, which was significantly higher than that of nearly all HEGs reported in the literature (Figure 2(e)). More importantly, the integrated *G.s*-PSII HPEG was able to generate electricity by harvesting both ambient latent heat and solar energy and directly power electronic devices in actual weather conditions.

To explain the excellent performances of all-biobased systems, the underlying mechanisms of electricity generation by *G.s*-PSII HPEGs were further studied and discussed in detail. Experimental results demonstrated that the improvement of output power was mainly attributable to the photovoltaic effect induced by photosensitization of *G.s*-PSII hybrid film, which created an electric field (*E*_P_) in the HPEGs and simultaneously increased the diffusion current. Under light illumination, PSII particles behaved as photosensitizers with active centers (P680) that were excited to generate electron-hole pairs [22]. Holes at PSII were effectively quenched by photoexcited electrons at surfaces of *G.s* cells due to the photosensitization of *G.s* via OM c-Cyts on cell membranes [24, 40]. Therefore, a photovoltaic electric field (*E*_P_) was formed from the holes at c-Cyt to the electrons of PSII (Figure 4(f)) in a direction perpendicular to the film surface. In addition, the hybridization between PSII and *G.s* facilitated the migration of photoelectrons and the separation of electron-hole pairs, which was beneficial to strengthen *E*_P_. Meanwhile, a hydrovoltaic electric field (*E*_H_) was formed in HPEGs, which originated from the abundant hydrophilic groups of hybrid film via water adsorption from ambient moisture [9, 10]. The accumulated water dissociated surface -COOH groups to release cations [10, 15], which then diffused into the interior of the hybrid film to create an ion concentration gradients across the hybrid film to form *E*_H_ (Figure S19). *E*_H_ and *E*_P_ were in the same direction within the system of *G.s*-PSII HPEG, thereby forming a superimposed total electric field (*E*_total_) for the improvement of output power.

**Figure 6:**
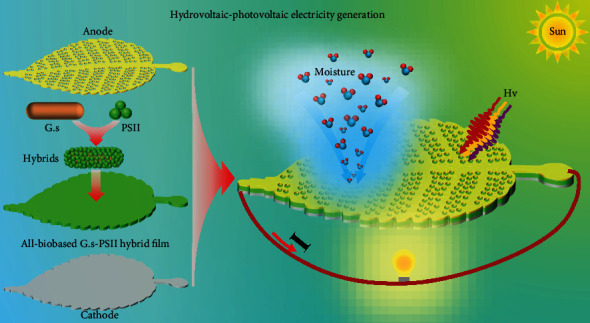
Schematic diagram of hydrovoltaic-photovoltaic electricity generation by simultaneously harvesting ambient energy from moisture and sunlight.

Specifically, the total output power (1.23 *μ*W) of an HPEG was significantly higher than the sum of a single hydro- (0.45 *μ*W) and photovoltaic (2.0 × 10^−4^ *μ*W) power generation (Figure 5(c)). It was therefore believed that a synergic effect existed between hydrovoltaic and photovoltaic electricity generation in *G.s*-PSII HPEGs. As shown, the photovoltaic power of *G.s*-PSII HPEGs was increased at a higher relative humidity (Figure 5(c)). The increment (0.78 *μ*W) of output power from dark to light at 90%RH was significantly higher than that at 10%RH (1 × 10^−4^ *μ*W) and 50%RH (0.06 *μ*W), which was in accordance with the changes in corresponding voltages and currents (Figure S25). As evidenced by electrochemical impedance spectra (EIS), the increase of relative humidity was beneficial to reducing film resistance of the *G.s*-PSII HPEG (Figure 5(d)). This was mainly due to the improved separation of photoexcited electron-hole pairs assisted by water molecules [31, 41], in a way that charge transfer and diffusion resistance were reduced by increasing mobile ions and electrons [42, 43]. Based on the above results, the coupling effect of hydrovoltaic and photovoltaic electricity generation of *G.s*-PSII HPEG was originated from simultaneously harvesting the energy of ambient moisture and sunlight (Figure 6).

In summary, all-biobased *G.s*-PSII HPEGs exhibited a broadly workable range of ambient humidity (10~90%RH) under full-spectrum light, which was promising for potential applications in most parts of the world. In addition, these HPEGs generated considerable output electricity under a relatively low light intensity (0.5~1.5 mW/cm^2^) in comparison to other HPEGs (100~200 mW/cm^2^) documented in the literature [18, 44], which further boosted the all-weather applications of *G.s*-PSII HPEGs. In addition, the maximum power density (1.24 W/m^2^) was generated under a light intensity of 100 mW/cm^2^, surpassing all HEGs reported hitherto. Considering the abundant and available resource of *G.s* and PSII in nature, we believe that all-biobased *G.s*-PSII HPEGs provide a feasible option for electricity generation via sustainable and green energy harvesting from ambient moisture and sunlight.

## 4. Materials and Methods

### 4.1. Preparation of PSII Particles

PSII particles were extracted from spinach leaves purchased from a local market through a modified procedure from previously reported methods [26, 27]. Briefly, deveined spinach leaves were well mixed by a low-speed blender in a mixed solution of 50 mM phosphate buffer solution (PBS) and 200 mM NaCl (pH = 7.8). After filtration with a 16-layered gauze, the green suspension was centrifuged (3,000 × *g* for 5 min) and resuspended in the above mixed solution. The mixed suspension was then centrifuged at 3,000 × *g* for 30 s, and the supernatant was recentrifuged at 3,000 × *g* for 5 min. Afterwards, the preparation was suspended in 10 mM NaCl and centrifuged at 3,000 × *g* for 5 min, and the supernatant was centrifuged (12,000 × *g* for 10 min) for collecting chloroplasts. To obtain PSII particles, the chloroplasts were suspended in a mix medium of 50 mM NaCl, 50 mM PBS, and 300 mM sucrose (pH = 6.8). The ratio of chlorophyll to Triton X-100 solution (20%*w*/*v*) was adjusted to 1 : 25 and stirred for 30 min. The mixed suspension was centrifuged at 5,000 × *g* for 10 min, and the supernatant was further centrifuged at 26,000 × *g* for 30 min. The precipitate was collected and resuspended in Tricine/NaOH buffer (10 mM, pH = 7.6) and centrifuged at 26,000 × *g* for 30 min, followed by washing with ultrapure water and stored at 4°C for further use.

### 4.2. Fabrication of *G.s*-PSII HPEGs

*G.s* used in this study was cultured in mineral medium at ~30°C under anaerobic atmosphere (N_2_/CO_2_ = 80/20), as described in a previous study [45]. The mineral medium contained fumarate (as an electron acceptor), acetate (as an electron donor), and other ingredients as summarized in Tables S3 and S4 in the Supplementary Materials. As-prepared PSII particles were injected into *G.s* suspensions when the *G.s* reached exponential phase (OD_600_~0.6). The PSII particles would couple to *G.s* surfaces to form *G.s*-PSII hybrids after 24 h, and then, the hybrids were centrifuged at 6,000 × *g*, collected, and resuspended in ultrapure water. A certain amount of suspension of *G.s*-PSII hybrids was dropped into a fixed model on an ITO glass slide and dried at 25°C in dark. After drying naturally, a *G.s*-PSII hybrid film was successfully prepared. Empirically, a 150 *μ*L/cm^2^*G.s*-PSII suspension (10 mg/mL) yielded a film thickness of ~30 *μ*m. A 100-mesh titanium electrode (1.0 × 1.0 cm^2^) was fixed on the top in a way that the *G.s*-PSII hybrid film was sandwiched between the titanium mesh and ITO glass slide. Films of single component PSII particles or *G.s* cells were fabricated in the same procedure and used as references for electricity generation tests. In addition, glassy carbon, Au, and Cu electrodes were selected to fabricate *G.s*-PSII HPEGs in unveiling the effect of redox reactions of electrode materials on the performance of electricity generation. These electrode materials were purchased from the online shopping platform (Taobao, China).

### 4.3. Extraction of OM c-Cyts

Extraction of OM c-Cyts was performed using a previously reported method [46]. Briefly, *G.s* cells were sheared in a low-speed blender at 4°C for 3 min. The cell suspension was centrifuged at 8,000 × *g* for 30 min (4°C) to obtain a supernatant. The OM c-Cyts in the supernatant were separated by 3 kDa Molecular Weight Cut-Off (MWCO) Spin Filter at 4,900 × *g* (4°C) and identified by UV-vis absorption spectra [47]. The proteinase K treatment of the *G.s* cells was conducted by the reported method [48].

### 4.4. Characterizations of *G.s*-PSII HPEGs

Morphology of *G.s*-PSII hybrid films was characterized by camera (D7500, Nikon, Japan), SEM (SU8010, Hitachi, Japan), and TEM (Talos F200X, FEI, USA) with an EDS. *G.s*-PSII hybrids were stained with the SYTO 9 stain of LIVE/DEAD BacLight Bacterial Viability Kit (Invitrogen, CA). Cellular localization of SYTO 9 fluorescence was determined by CLSM (LSM880, Carl Zeiss, Germany) with an excitation and detection wavelength of 488 nm and 498-550 nm, respectively. Meanwhile, a detection wavelength of 610-700 nm was used to observe the autofluorescence of chlorophyll [29]. Size distribution and zeta potential were measured by nanoparticle size potentiometer (Zetasizer Nano S, Malvern, UK). UV-vis absorption and diffuse reflectance spectra (DRS) were recorded by a UV spectrometer (UV2600, Shimadzu, Japan). Contact angles (CAs) were tested by a goniometer (OCA20, DataPhysics, Germany). Ion conductivity was measured by conductivity meter (DDSJ-308F, INESA, China). Element analysis and VB XPS test were performed by X-ray photoelectron spectroscopy (ESCALAB 250XI, Thermo Fisher, USA). PL emission and delay spectra were recorded by photoluminescence spectrometer (FLS980, Edinburgh Instruments, UK). The average lifetime of PL decay was calculated via the equation *τ* = ∑(*A*_n_*τ*_n_^2^)/∑(*A*_n_*τ*_n_) [32].

### 4.5. Electrical Measurements

Electricity tests were conducted with an electric measurement system (PalmSens4, PALMSENS, Netherlands). The current of circuit parameters was set to zero during output voltage tests. Correspondingly, the voltage of circuit parameters was set to zero during output current tests. A full-spectrum light-emitting diode (LED) was utilized as the light source. EIS tests were performed by PalmSens4 in a frequency range of 10^−2^~10^6^ Hz. Photocurrent tests were conducted by electrochemical workstation (CHI 660E, Chenhua, China) with a light intensity of 1.0 mW/cm^2^ and 0.4 V (vs. SCE) as previously described [49]. Leaf-shaped *G.s*-PSII hybrid films were prepared on PET-ITO, and then, porous leaf-shaped electrodes were fixed on top of the dried films to fabricate leaf-shaped HPEGs. Twelve leaf-shaped HPEGs were connected in series to form an integrated HPEG. The output power of integrated HPEGs was tested through lighting an electronic ink screen in all weathers, including cloudy, rainy, and sunny days. The maximum power density (*P*_max_) was controlled by the maximum rectangular area voltage within the range of current-voltage (*I-V*) curves or estimated by (*V*_oc_ · *I*_sc_)/4 [9, 50].

## Data Availability

Data supporting the findings of this study are available in the main text or the Supplementary Materials.

## Supplementary Materials

Supplementary Materials Figure S1: TEM image of PSII particles. Figure S2: SEM image of *G.s* cell. Figure S3: photograph of PSII film, *G.s* film, and *G.s*-PSII hybrid film (effective area = 1.0 × 1.0 cm^2^) on ITO glass slides (2.0 × 1.4 cm^2^). Figure S4: contact angle measurements of the PSII film, *G.s* film, and *G.s*-PSII hybrid film. Figure S5: the effects of only light on the electric output of *G.s*-PSII HPEG. Figure S6: the influence of light illumination on temperature and activity of films. Figure S7: verifications of electricity generation. Figure S8: the effect of electrode materials on electricity generation performance of the *G.s*-PSII HPEG. Current output of the HPEG prepared by (a) glassy carbon, (b) Au, and (c) Cu electrodes (film size = 1.0 × 1.0 cm^2^). Figure S9: the effect of influence factors on electricity generation performance. (a) The effect of PSII content (wt%) in the *G.s*-PSII hybrid film on electricity generation performance. (b) The effect of film thickness on electricity generation performance. The different letters represent statistically significant difference (*P* < 0.05). Figure S10: voltage, current, and output power density of the HPEG with variable electric resistances. Figure S11: the effect of film size on electricity generation performance. (a) The effect of film size on voltage and current output. (b) The effect of film size on power and power density output. Figure S12: (a) the relationship between the light intensity and power density of HPEG (1 × 1 mm^2^) under 90%RH and the temperature of HPEG under different light intensities. (b) Output power density of HPEG under the different temperatures reaching by hot plate according to same photothermal temperatures. Figure S13: the electronic ink screen (15 × 15 cm^2^) is disconnected to the integrated HPEG. Figure S14: PL emission spectra of the pure PSII particles, the pure *G.s* cells, and the *G.s*-PSII hybrids at an excitation wavelength of 680 nm. Figure S15: Tauc plots of PSII and *G.s* from UV-vis diffuse reflectance spectra with calculated bandgap energy (*E*_g_). Figure S16: photosensitive characteristics of *G.s* and the extracted OM c-Cyts. (a) Valence band XPS spectra. (b) Tauc plots from UV-vis diffuse reflectance spectra with calculated bandgap energy (*E*_g_), the inset plot is the energy band diagrams. Figure S17: the effect of OM c-Cyt removal in *G.s*. on light response of the HPEG. (a) UV-vis absorption spectra of *G.s* and proteinase K-treated *G.s* at 90%RH. (b) Voltage output tests of the *G.s*-PSII and the proteinase K-treated *G.s*-PSII HPEGs under darkness and light at 90%RH. Figure S18: the effect of OM c-Cyt addition in *G.s*. on light response of the *G.s*-PSII HPEG. (a) UV-vis absorption spectra of *G.s* and *G.s* with OM c-Cyt addition. (b) Voltage output tests of the *G.s*-PSII and the (*G.s*+OM c-Cyt)-PSII HPEGs under darkness and light at 90%RH. Figure S19: the mechanism of hygroelectricity generation for the *G.s*-PSII HPEG. Figure S20: moisture adsorption in peel-off films. The existence of moisture gradient can be further verified by following analysis. If a moisture gradient existed (i.e., the local adsorption would be dependent on its depth from the interface), then exposing both interfaces would yield increased moisture adsorption. Figure S21: XPS oxygen spectra of the *G.s*-PSII hybrid film. Figure S22: schematic of the energy transformation in hygroelectricity. Figure S23: the effect of RHs on output voltage and current of the (a) PSII- and (b) *G.s-*based devices under darkness. Figure S24: ion conductivity and zeta potential of PSII film, *G.s* film, and *G.s*-PSII hybrid film. The different letters represent statistically significant difference (*P* < 0.05). Figure S25: effect of different RHs on electricity generation of the HPEG under darkness and light, respectively. Effect of different RHs on (a) power output, (b) voltage output, and (c) current output. The red letter is the increase of the value, simultaneously harvesting ambient energy from moisture and sunlight. Table S1: summary and comparison of sustained HEGs. Table S2: fit parameters for the PL decay curves. Table S3: NBAF media for *Geobacter sulfurreducens*. Table S4: 100× NB Salts Solution, NB Mineral Elixir Solution, and DL Vitamins Solution of NBAF media of *Geobacter sulfurreducens*. Movie S1: the integrated HPEG directly powered the electronic ink screen under cloudy sky. Movie S2: the integrated HPEG directly powered the electronic ink screen under rainy day. Movie S3: the integrated HPEG directly powered the electronic ink screen under sunlight.
