# Erratum to “All-Biobased Hydrovoltaic–Photovoltaic Electricity Generators for All-Weather Energy Harvesting”

**DOI:** 10.34133/research.0454

**Published:** 2024-08-26

**Authors:** Guoping Ren, Qichang Hu, Jie Ye, Andong Hu, Jian Lü, Shungui Zhou

**Affiliations:** ^1^ College of Resources and Environment, Fujian Agriculture and Forestry University, Fuzhou, China.; ^2^ College of Mechanical and Electrical Engineering, Fujian Agriculture and Forestry University, Fuzhou, China.

In the Research Article, “All-Biobased Hydrovoltaic–Photovoltaic Electricity Generators for All-Weather Energy Harvesting” [1], the publisher inadvertently introduced an error in Fig. 2F. The power density on the *y*-axis was incorrectly labeled as (mW/cm^2^) instead of the correct (mW/m^2^). Figure 2F has now been corrected in the PDF and HTML (full text). The layout of the figure panels has also been updated for better readability.

**Fig. 2.**
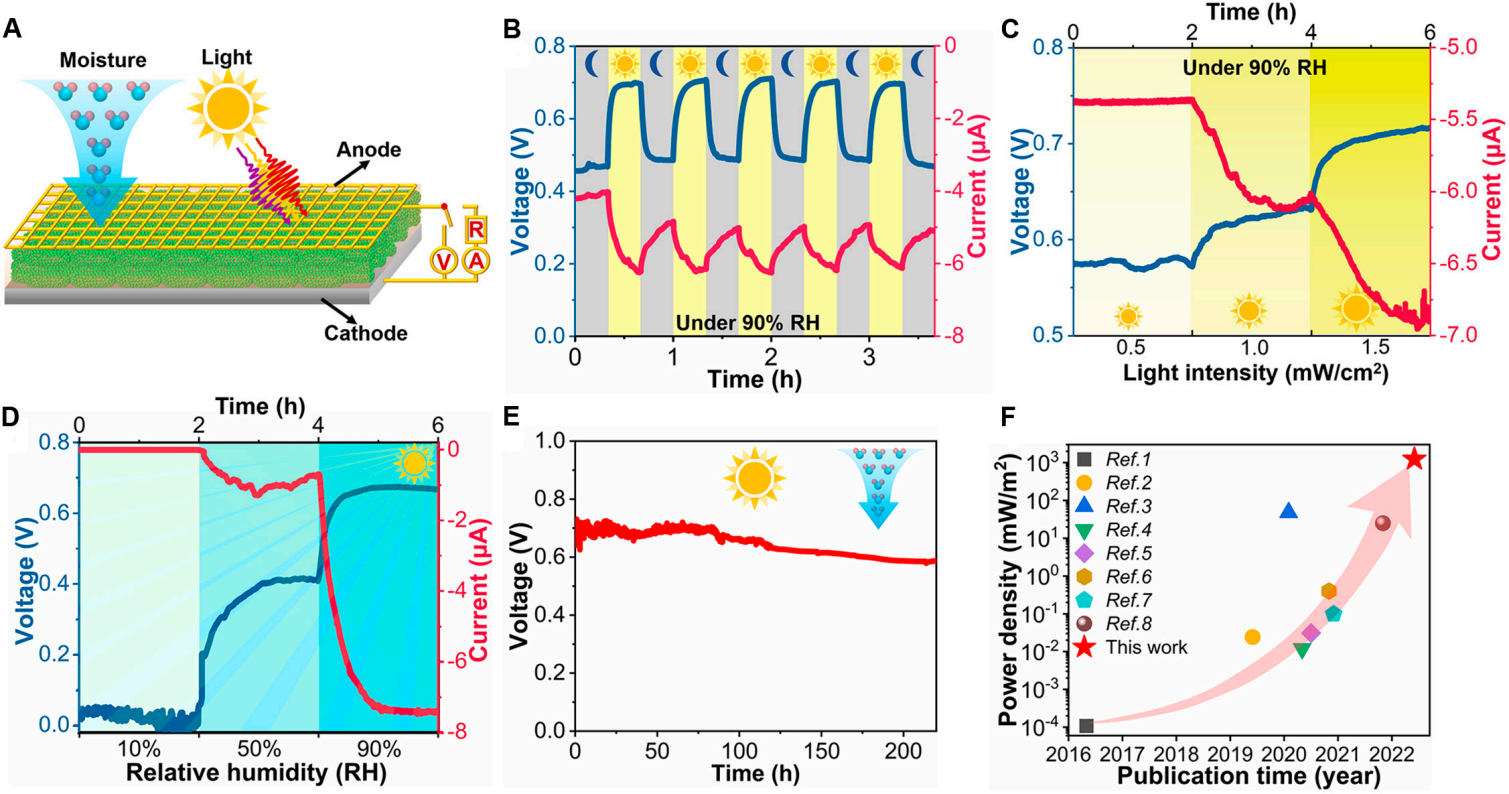
Electric output performance of the *G.s*-PSII HPEG. (A) Schematic diagram of the HPEG to generate electricity. (B) Electric output of the HPEG responds to intermittent light–darkness at 90%RH, where the gray and yellow backgrounds are in darkness and light, respectively (25 ± 2) °C. (C) Continuous voltage and current measurements under different light intensities (0.5, 1.0, and 1.5 mW/cm^2^) at 90%RH (25 ± 2) °C. (D) Continuous voltage and current measurements at different RHs (10%, 50%, and 90%RH) under a light intensity of 1.5 mW/cm^2^ (25 ± 2) °C. (E) Long-time test of voltage under light at 90%RH and (25 ± 2) °C. (F) A comparison of the power density of this current device with those of the sustainable hygroelectricity generators in the literature (Table S1).

## References

[1] Ren G, Hu Q, Ye J, Hu A, Lü J, Zhou S. All-biobased hydrovoltaic–photovoltaic electricity generators for all-weather energy harvesting. Research (Wash D C). 2022;2022: Article 9873203. 36082209 10.34133/2022/9873203 PMC9429978

