# Erratum to “Enhanced Effects of Intermittent Fasting by Magnetic Fields in Severe Diabetes”

**DOI:** 10.34133/research.0501

**Published:** 2024-10-11

**Authors:** Ying Wang, Chuanlin Feng, Biao Yu, Junjun Wang, Weili Chen, Chao Song, Xinmiao Ji, Ruowen Guo, Guofeng Cheng, Hanxiao Chen, Xinyu Wang, Lei Zhang, Zhiyuan Li, Jialiang Jiang, Can Xie, Haifeng Du, Xin Zhang

**Affiliations:** ^1^ High Magnetic Field Laboratory, CAS Key Laboratory of High Magnetic Field and Ion Beam Physical Biology, Hefei Institutes of Physical Science, Chinese Academy of Sciences, Hefei, Anhui, China.; ^2^ Science Island Branch of Graduate School, University of Science and Technology of China, Hefei, Anhui, China.; ^3^ NHC Key Laboratory of Study on Abnormal Gametes and Reproductive Tract, Anhui Medical University, Hefei, Anhui, China.; ^4^ Institutes of Physical Science and Information Technology, Anhui University, Hefei, Anhui, China.; ^5^ Medical Research Council (MRC) Protein Phosphorylation and Ubiquitylation Unit, School of Life Sciences, University of Dundee, Dundee, UK.

In the Research Article “Enhanced effects of intermittent fasting by magnetic fields in severe diabetes”, an error was inadvertently introduced during the production process [1]. The unit of measurement in Fig. 1B is “KGs”, not “kg”. The publisher apologizes for this error, which is corrected in the figure below.

**Fig. 1.**
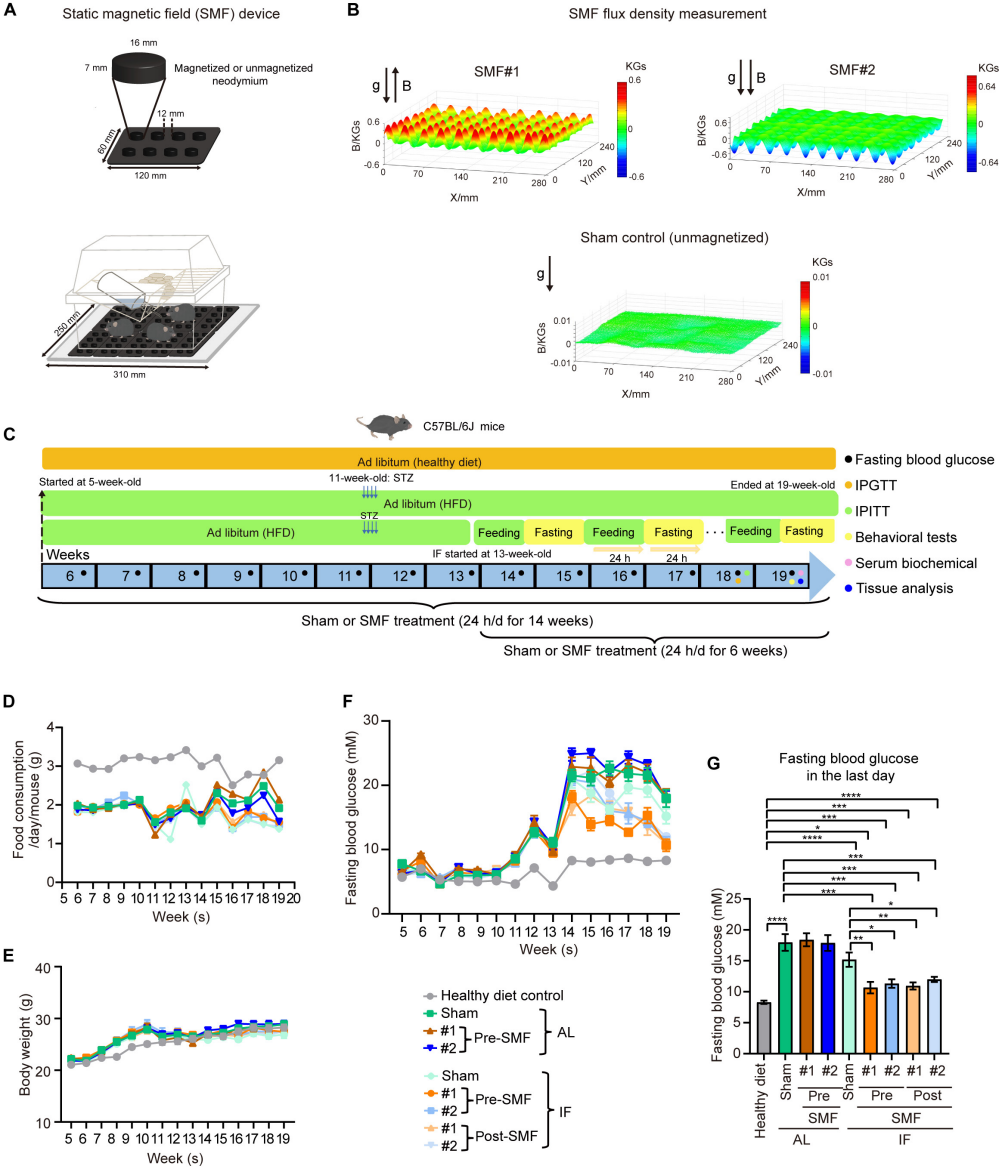
SMFs promote the blood glucose reduction effects of IF in moderate diabetic mice. (A) The SMF devices. (B) Magnetic field distribution in the mouse exposure area, 8 mm above the magnetized (SMF#1 and SMF#2) or unmagnetized (sham control) plates. (C) Experimental design. HFD + STZ-induced C57BL/6J diabetic mice were used. (D) Food consumption of mice (*n* = 8 to 10 mice per group). (E) Body weight of mice (*n* = 8 to 10 mice per group). (F) Fasting blood glucose curves of mice [*n* = 8 to 10 mice per group, for (D) to (F) weeks indicating the mouse age]. Fasting time, 6 h. (G) Fasting blood glucose of mice on the last day of the experiment (*n* = 8 to 10 mice per group). All data are presented as means ± SEM and analyzed by GraphPad Prism 9.0. *P < 0.05, **P < 0.01, ***P < 0.001, and ****P < 0.0001, 2-tailed Student’s *t* test (G).
