# Erratum to “Inhibition of ALOX12–12-HETE Alleviates Lung Ischemia–Reperfusion Injury by Reducing Endothelial Ferroptosis-Mediated Neutrophil Extracellular Trap Formation”

**DOI:** 10.34133/research.0531

**Published:** 2024-11-08

**Authors:** Chongwu Li, Peigen Gao, Fenghui Zhuang, Tao Wang, Zeyu Wang, Guodong Wu, Ziheng Zhou, Huikang Xie, Dong Xie, Deping Zhao, Junqi Wu, Chang Chen

**Affiliations:** ^1^ Department of Thoracic Surgery, Shanghai Pulmonary Hospital, School of Medicine, Tongji University, Shanghai, China.; ^2^ Shanghai Engineering Research Center of Lung Transplantation, Shanghai, China.; ^3^ Department of Thoracic and Cardiovascular Surgery, The First Affiliated Hospital of Chongqing Medical University, Chongqing, China.; ^4^ Department of Pathology, Shanghai Pulmonary Hospital, School of Medicine, Tongji University, Shanghai, China.

In the Research Article “Inhibition of ALOX12–12-HETE Alleviates Lung Ischemia–Reperfusion Injury by Reducing Endothelial Ferroptosis-Mediated Neutrophil Extracellular Trap Formation”, the authors have identified 2 inadvertent errors in their figures [1].

In the upper right panel of Fig. 2A, the H&E image of *Alox12*-KO+Sham group was incorrect. Additionally, during the figure assembly process, the incorrect image of ACTB was used in Fig. 6F and H.

**Fig. 2.**
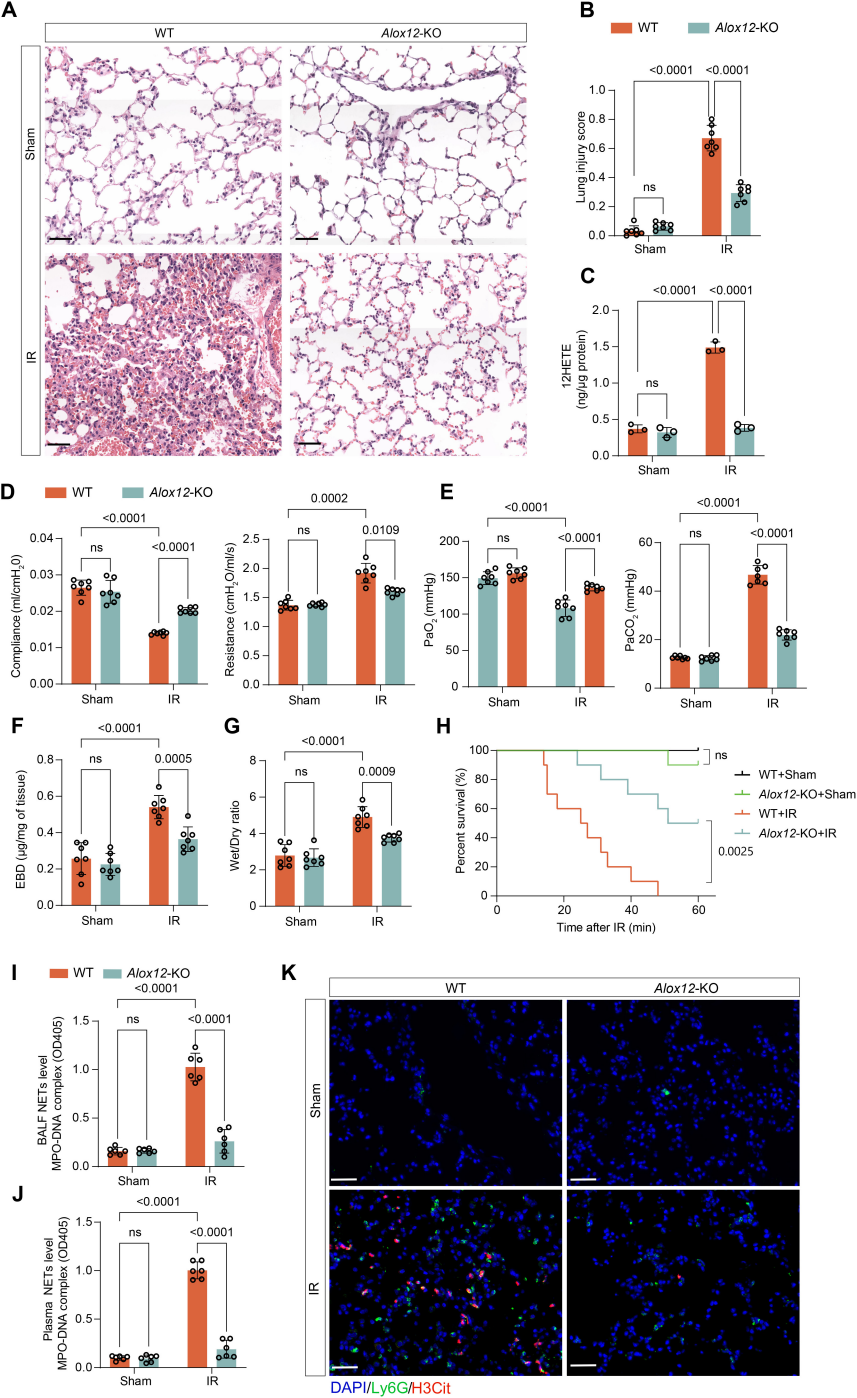
*Alox12*-KO alleviated lung injury, improved pulmonary function, prolonged survival, and reduced NET formation following lung IR. (A and B) H&E staining (A) and lung injury scores (B) showed significantly less lung damage in *Alox12*-KO mice compared to WT controls after IR. Scale bar, 50 μm; *n* = 7 in each group. (C) ELISA confirms a significant reduction in 12-HETE levels in the absence of *Alox12* after reperfusion; *n* = 3 in each group. (D and E) *Alox12*-KO mice exhibited better airway compliance, reduced airway resistance (D), higher PaO_2_, and lower PaCO_2_ (E) after IR, indicating preserved lung function; *n* = 7 in each group. (F and G) Evans blue dye extravasation (F) and wet/dry lung weight ratios (G) demonstrated reduced edema in *Alox12*-KO mice after reperfusion; *n* = 7 in each group. (H) Survival of mice depended solely upon the left lung (with right hilum ligated), showing that *Alox12*-KO mice had better survival rates compared with WT mice after lung IR; *n* = 10 in each group. (I to K) The level of NETs after reperfusion, measured by MPO-DNA complex in the BALF (I) and plasma (J), and immunofluorescence staining for H3Cit and Ly6G in lung tissues (K), was lower in *Alox12-*KO mice than in WT mice; *n* = 7 in each group. Scale bar, 50 μm. The data are presented as means ± SDs. Significance was examined with one-way ANOVA in (B) to (G), (I), and (J) and log-rank (Mantel–Cox) test in (H).

**Fig. 6.**
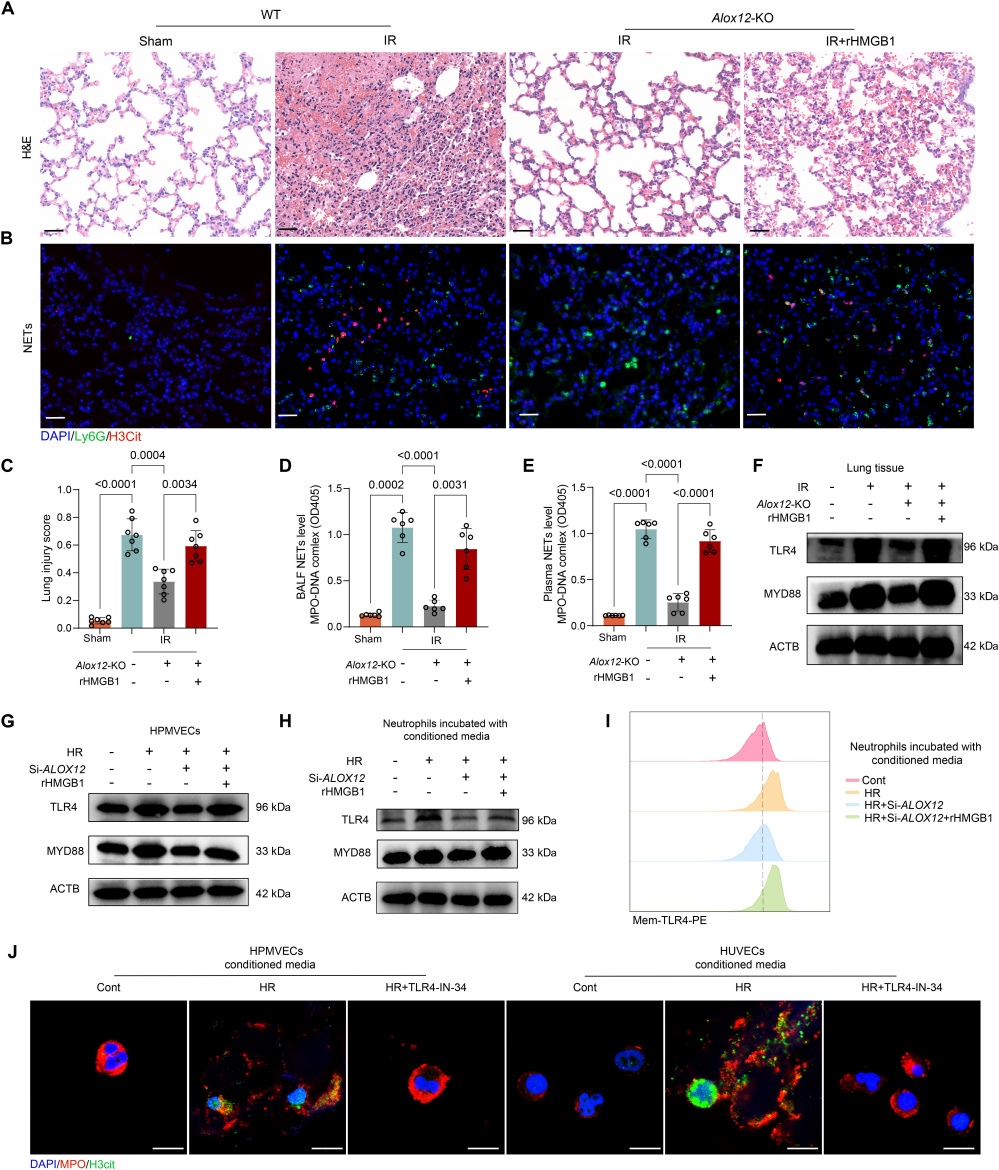
HMGB1 abrogated the protective effects of *Alox12* deficiency against IRI and activated the TLR4-MYD88 pathway to mediate NET formation. (A to E) rHMGB1 treatment reversed the inhibitory effects of *Alox12* deficiency on reperfusion-induced changes. H&E staining of left lung tissues (A) and lung injury scores (C) showed that the reduction in lung injury observed in *Alox12*-KO mice after reperfusion was reversed by the treatment with rHMGB1. Immunofluorescence staining for H3Cit and Ly6G in lung tissues (B), along with measurements of NET levels (MPO-DNA complex) in the BALF (D) and plasma (E), revealed that the reduction in NET formation in *Alox12*-KO mice after reperfusion was reversed by the treatment with rHMGB1. Scale bars, 50 μm (A) and (B); *n* = 7 in each group for (C) and *n* = 6 in each group for (D) and (E). (F and G) In both lung tissues after IRI (F), and HPMVEC cells subjected to HR (G), the protein levels of TLR4 and MYD88 increased following injury but decreased with *Alox12* deficiency. This reduction was reversed by rHMGB1 treatment. (H and I) Immunoblotting and flow cytometry analyses revealed that both total protein levels of TLR4 and MYD88 (H), as well as TLR4 membrane levels (I) in neutrophils, increased after incubation with conditioned medium from HR-induced endothelial cells. These levels decreased with *ALOX12* knockdown but were restored by rHMGB1 treatment. (J) TLR4-IN-34, a TLR4 inhibitor, prevented NET formation in neutrophils exposed to conditioned medium from HR-treated endothelial cells. Scale bar, 10 μm. The data are presented as means ± SDs. Significance was examined by one-way ANOVA in (C) to (E).

Figures 2 and 6 have now been corrected in the original publication. Although these errors did not affect the main conclusions, the authors sincerely apologize for the inconvenience.
