# Supplementary material for: All-Biobased Hydrovoltaic-Photovoltaic Electricity Generators for All-Weather Energy Harvesting
Source: Research (Wash D C). 2022 Aug 20;2022:9873203. doi: 10.34133/2022/9873203 (PMC9429978; doi:10.34133/2022/9873203)
Supplement: Supplementary Materials — Figure S1: TEM image of PSII particles. Figure S2: SEM image of G.s cell. Figure S3: photograph of PSII film, G.s film, and G.s-PSII hybrid film (effective area = 1.0 × 1.0 cm2) on ITO glass slides (2.0 × 1.4 cm2). Figure S4: contact angle measurements of the PSII film, G.s film, and G.s-PSII hybrid film. Figure S5: the effects of only light on the electric output of G.s-PSII HPEG. Figure S6: the influence of light illumination on temperature and activity of films. Figure S7: verifications of electricity generation. Figure S8: the effect of electrode materials on electricity generation performance of the G.s-PSII HPEG. Current output of the HPEG prepared by (a) glassy carbon, (b) Au, and (c) Cu electrodes (film size = 1.0 × 1.0 cm2). Figure S9: the effect of influence factors on electricity generation performance. (a) The effect of PSII content (wt%) in the G.s-PSII hybrid film on electricity generation performance. (b) The effect of film thickness on electricity generation performance. The different letters represent statistically significant difference (P < 0.05). Figure S10: voltage, current, and output power density of the HPEG with variable electric resistances. Figure S11: the effect of film size on electricity generation performance. (a) The effect of film size on voltage and current output. (b) The effect of film size on power and power density output. Figure S12: (a) the relationship between the light intensity and power density of HPEG (1 × 1 mm2) under 90%RH and the temperature of HPEG under different light intensities. (b) Output power density of HPEG under the different temperatures reaching by hot plate according to same photothermal temperatures. Figure S13: the electronic ink screen (15 × 15 cm2) is disconnected to the integrated HPEG. Figure S14: PL emission spectra of the pure PSII particles, the pure G.s cells, and the G.s-PSII hybrids at an excitation wavelength of 680 nm. Figure S15: Tauc plots of PSII and G.s from UV-vis diffuse reflectance spe [file 9873203.f1.zip › Supporting Materials.docx]

**Supporting Materials**

All-Biobased Hydrovoltaic-Photovoltaic Electricity Generators for All-Weather Energy Harvesting

Short title: **Biobased Generator to Capture All-Weather Energy**

Guoping Ren^1†^, Qichang Hu^1,2†^, Jie Ye^1^, Andong Hu^1^, Jian Lü^1*^, Shungui Zhou^1*^

^1^ College of Resources and Environment, Fujian Agriculture and Forestry University, Fuzhou, China.

^2^ College of Mechanical and Electrical Engineering, Fujian Agriculture and Forestry University, Fuzhou, China

Correspondence should be addressed to Jian Lü; jian_lu_fafu@163.com and Shungui Zhou; sgzhou@fafu.edu.cn.

†These authors contributed equally to this work.


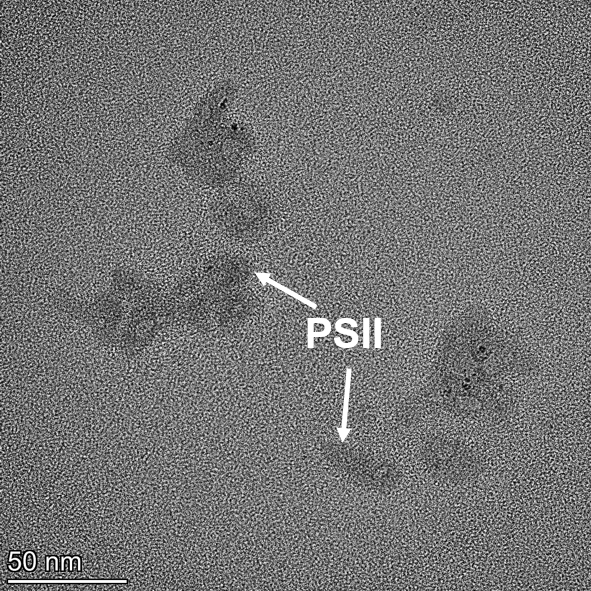


**Figure S1**. TEM image of PSII particles. PSII with particle sizes ranging from ca. 20 ~ 40 nm, as indicated from a transmission electron microscopy (TEM) image.


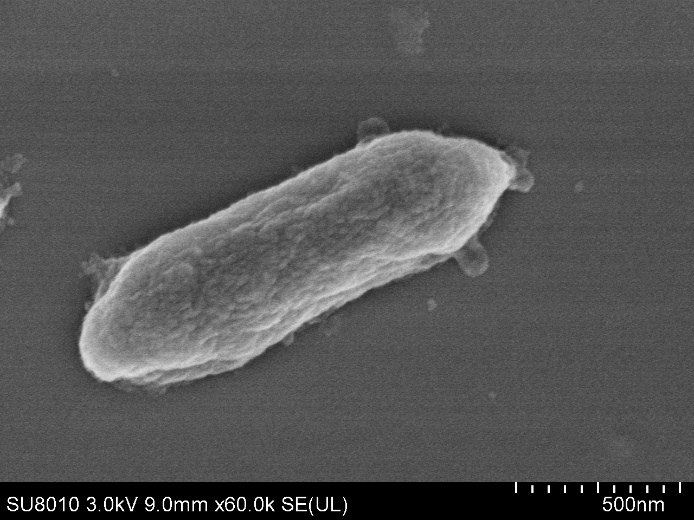


**Figure S2.** SEM image of *G.s* cell.


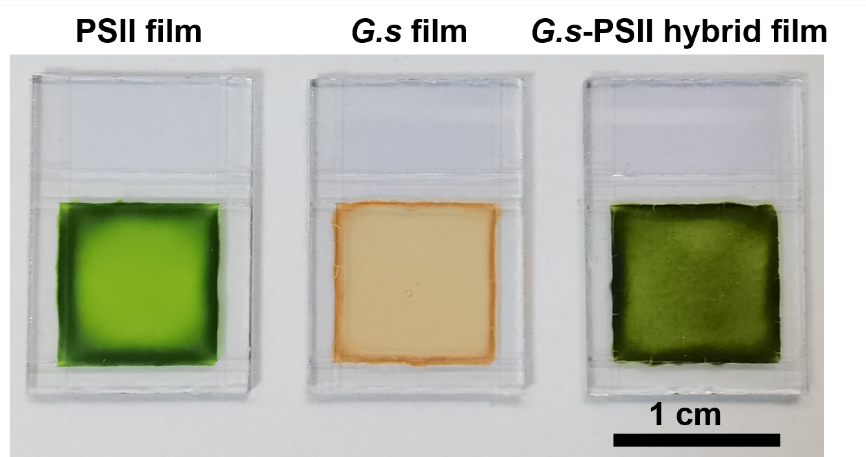


**Figure S3.** Photograph of PSII film, *G.s* film, and *G.s*-PSII hybrid film (effective area = 1.0 🞨 1.0 cm^2^) on ITO glass slides (2.0 🞨 1.4 cm^2^).


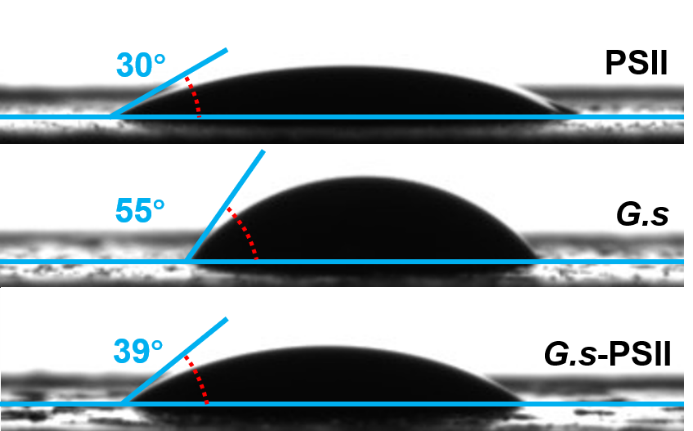


**Figure S4.** Contact angle measurements of the PSII film, *G.s* film, and *G.s*-PSII hybrid film.


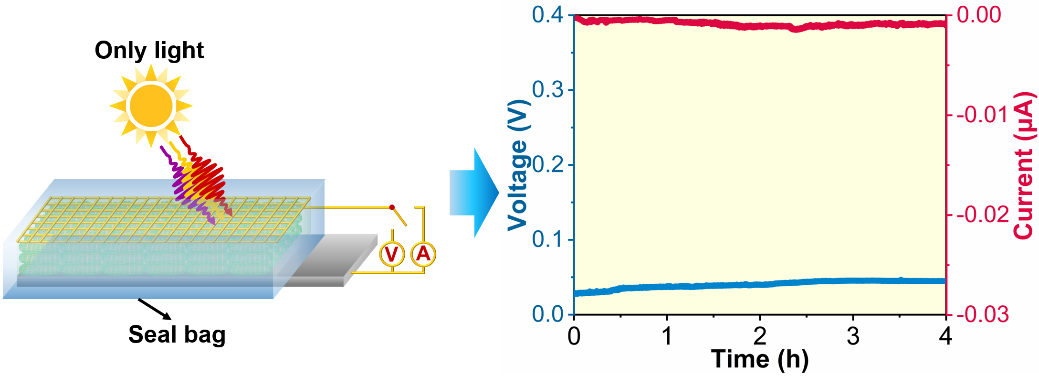


**Figure S5.** The effects of only light on the electric output of *G.s*-PSII HPEG. Schematic diagram and electric output of the *G.s*-PSII HPEG under light in a seal bag to resist the environmental moisture (~10%RH).


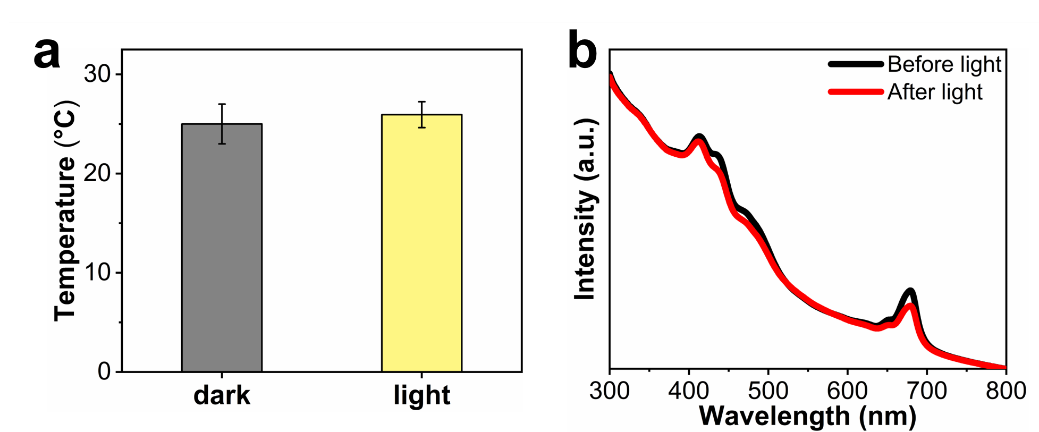


**Figure S6.** The influence of light illumination on temperature and activity of films. (a) The influence of light illumination (light intensity of 1.5 mW/cm^2^) on temperature. (b) UV-vis absorption spectra variations before and after light illumination (1.5 mW/cm^2^).


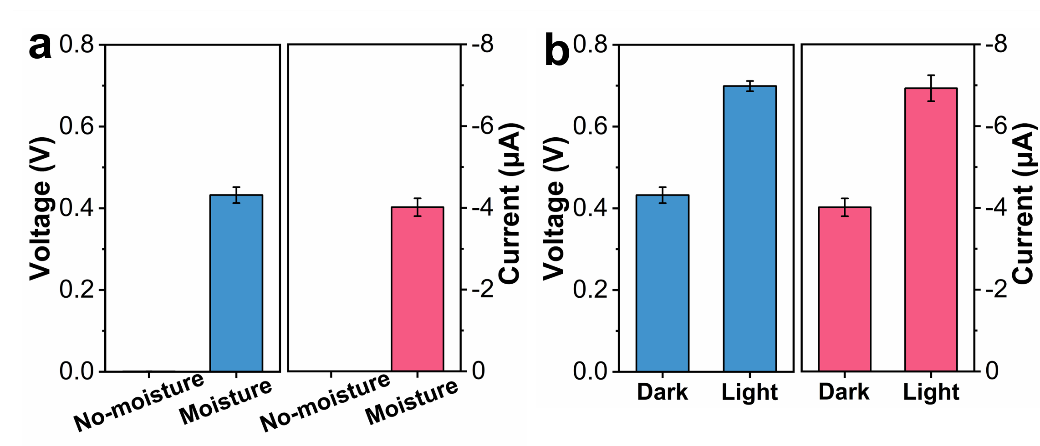


**Figure S7.** Verifications of electricity generation. (a) Voltage and current generations under no-moisture (under 99.99% nitrogen environment and without moisture water under dark condition) and moisture (at 90%RH under dark condition), respectively. (b) Voltage and current generations under dark and light (light intensity of 1.5 mW/cm^2^) at 90%RH, respectively.


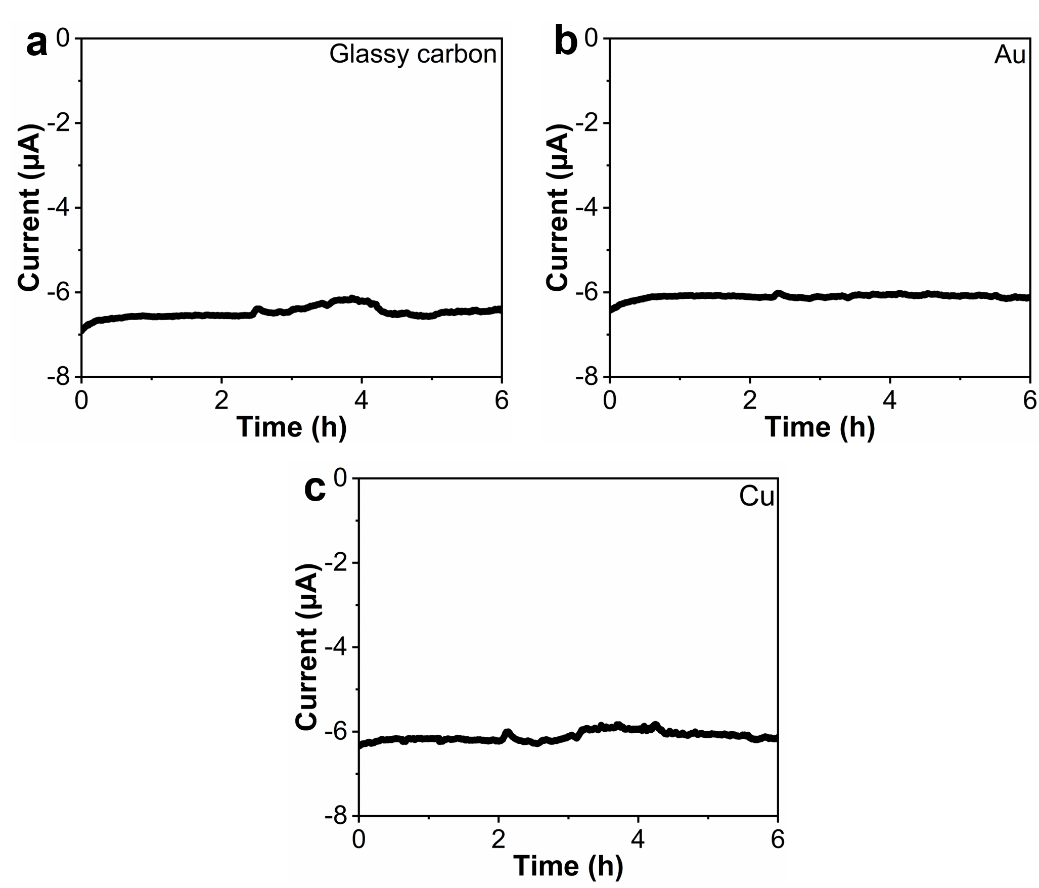


**Figure S8.** The effect of electrode materials on electricity generation performance of the *G.s*-PSII HPEG. (a) Current output of the HPEG prepared by glassy carbon, (b) Au and (c) Cu electrodes. (film size = 1.0🞨1.0 cm^2^).

In order to study the effect of redox reaction of electrode material on the electricity generation, three kinds of materials (glassy carbon, Au and Cu) were selected as the electrodes to fabricate the corresponding HPEGs. these HPEGs all could generate steady current under light at 90%RH, which were about 6.0 ~ 7.0 μA during 6 h. This result indicated the redox reaction has very little effect on electricity generation in HPEG.


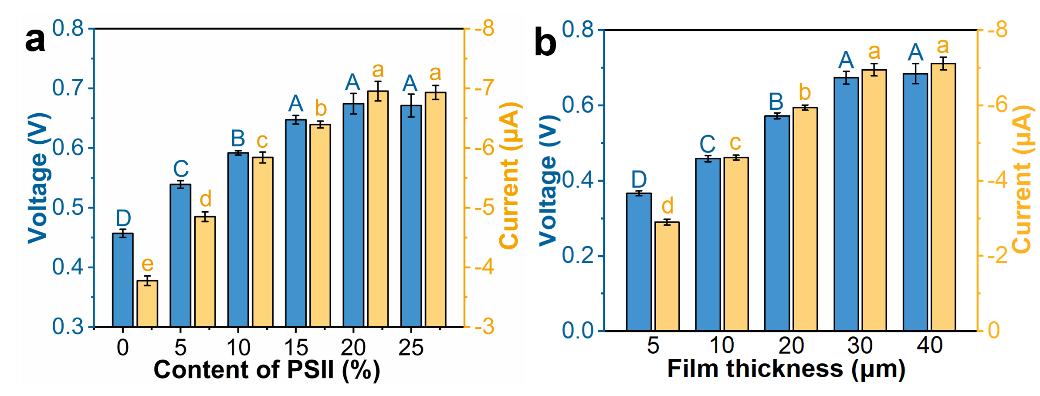


**Figure S9.** The effect of influence factors on electricity generation performance. (a) The effect of PSII content (wt%) in the *G.s*-PSII hybrid film on electricity generation performance. (b) The effect of film thickness on electricity generation performance. The different letters represent statistically significant difference (*P* < 0.05).


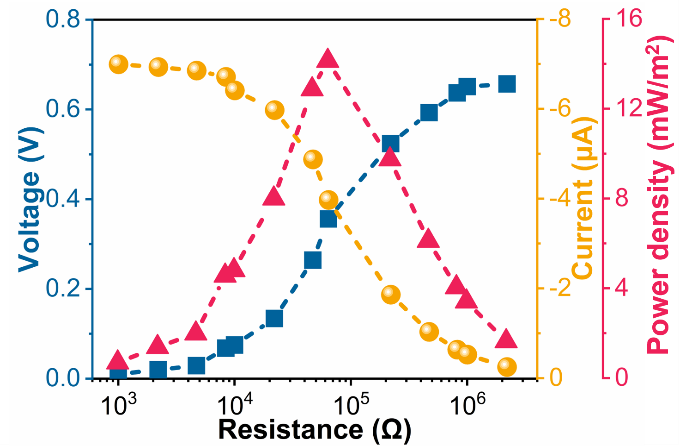


**Figure S10.** Voltage, current and output power density of the HPEG with variable electric resistances. Power density is calculated by (load voltage 🞨 load current)/(1.0 cm^2^).


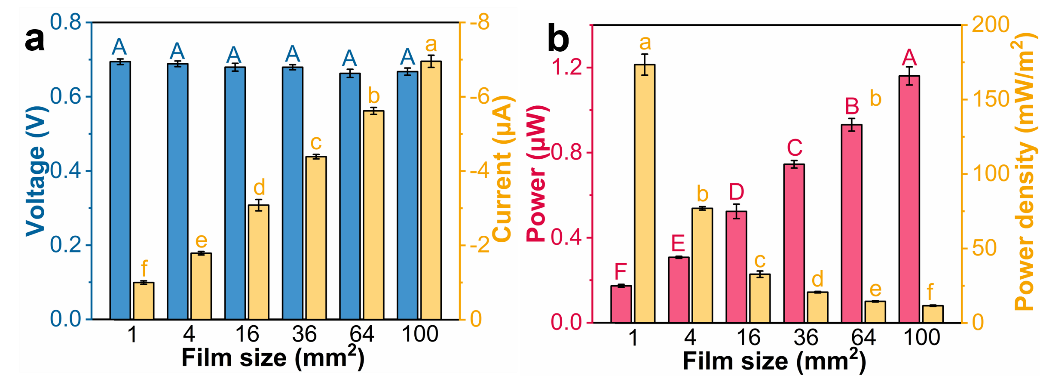


**Figure S11.** The effect of film size on electricity generation performance. (a) The effect of film size on voltage and current output. (b) The effect of film size on power and power density output. The areal power density is estimated by (*V_oc_*·*I_sc_*)/4. The different letters represent statistically significant difference (*P* < 0.05).


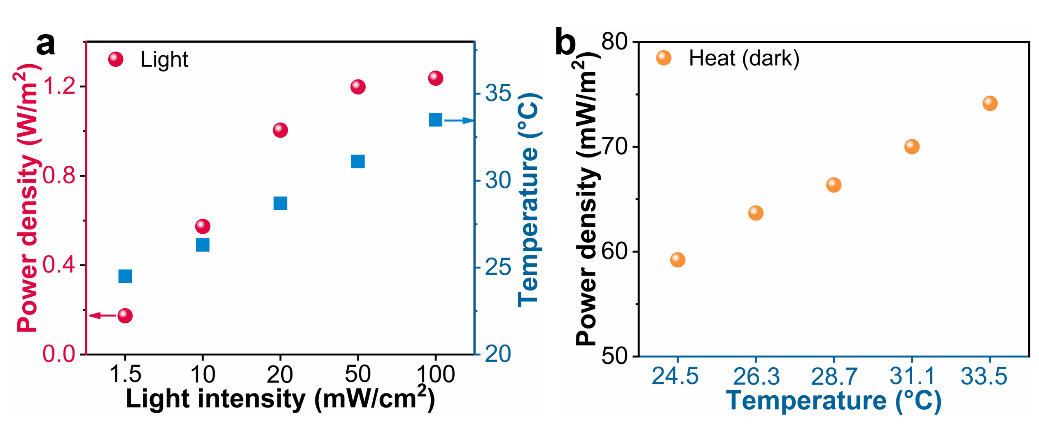


**Figure S12.** (a) The relationship between the light intensity and power density of HPEG (1 🞨 1 mm^2^) under 90%RH, and the temperature of HPEG under different light intensities. (b) Output power density of HPEG under the different temperatures reaching by hot plate according to same photothermal temperatures. This result indicated that higher temperature would improve output power density (b) of HPEG, but the increment was significantly lower than light-induced increase of power density (a).


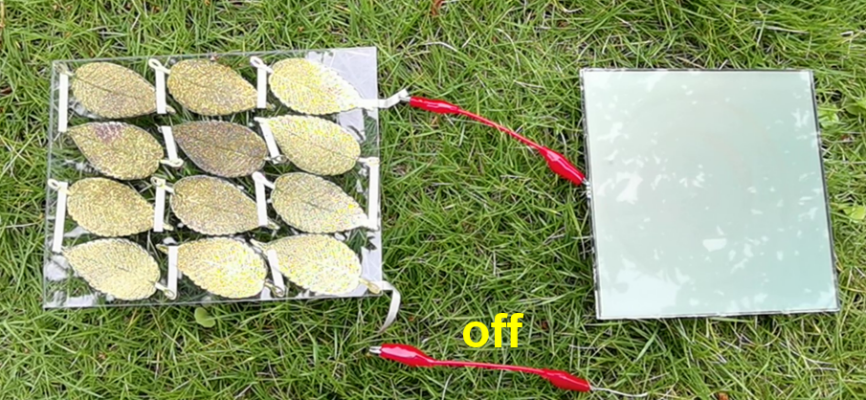


**Figure S13.** The electronic ink screen (15 🞨 15 cm^2^) is disconnected to the integrated HPEG.


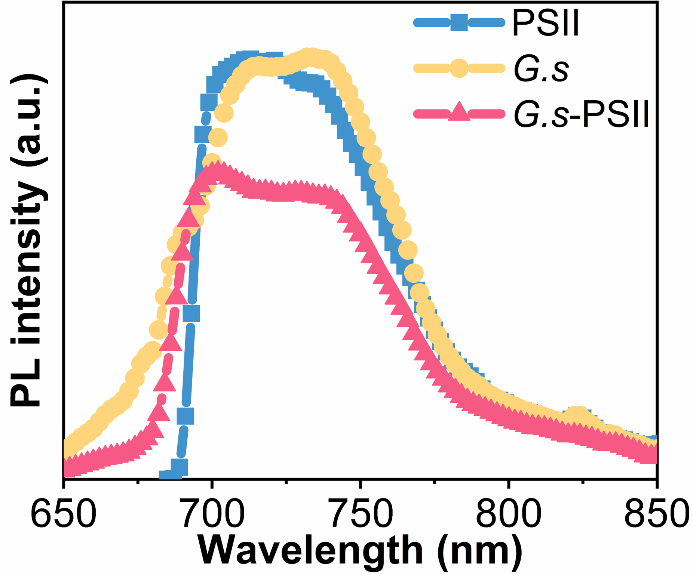


**Figure S14.** PL emission spectra of the pure PSII particles, the pure *G.s* cells and the *G.s*-PSII hybrids at an excitation wavelength of 680 nm.





**Figure S15.** Tauc plots of PSII and *G.s* from UV-vis diffuse reflectance spectra with calculated bandgap energy (*E*_g_).


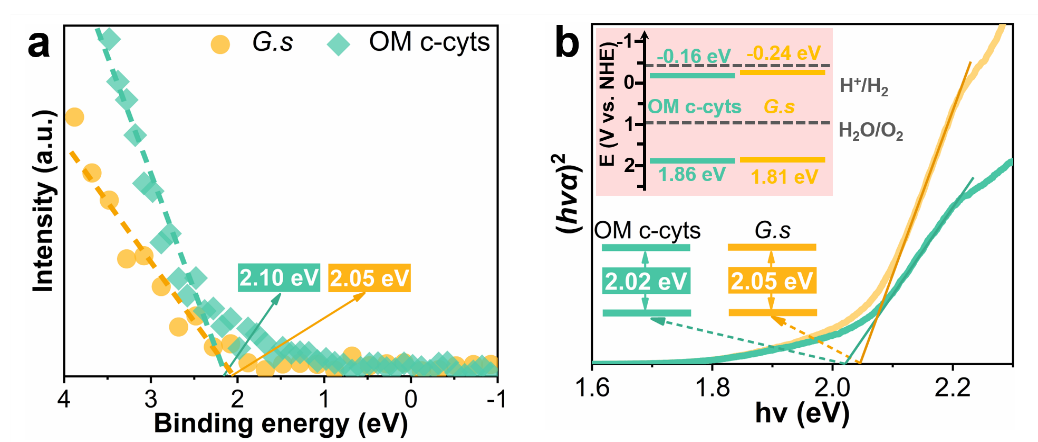


**Figure S16.** Photosensitive characteristics of *G.s* and the extracted OM c-Cyts. (a) Valence band XPS spectra. (b) Tauc plots from UV-vis diffuse reflectance spectra with calculated bandgap energy (*E*_g_), the inset plot is the energy band diagrams. The conduction band (CB) energy (*E*_CB_) was calculated based on the formula of *E*_CB_ = *E*_VB_ - *E*_g_. The band structures of *G.s* and PSII were estimated based on the formula of *E*_NHE_ = Φ - 4.44 + *E*_VB_ (*E*_NHE_: potential of normal hydrogen electrode; Φ (4.2 eV): electron work function of the XPS analyzer).


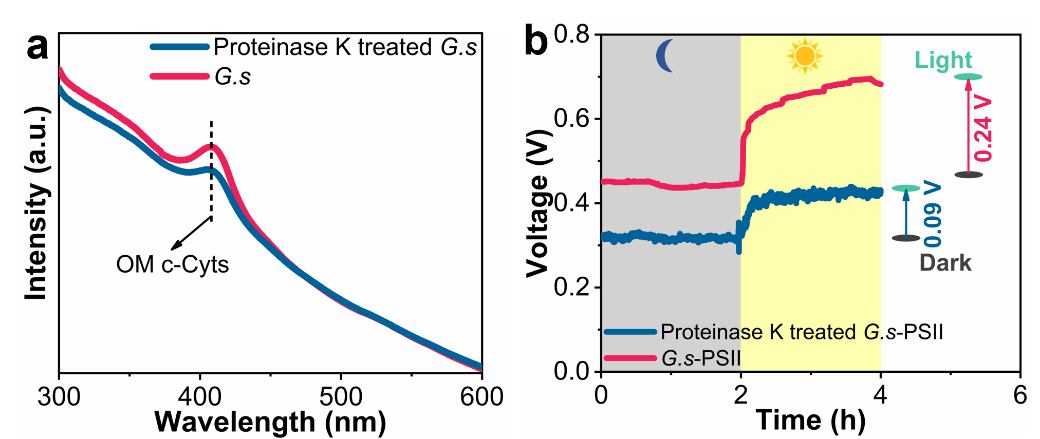


**Figure S17.** The effect of OM c-Cyts removal in *G.s.* on light-response of the HPEG. (a) UV-vis absorption spectra of *G.s* and proteinase K treated *G.s* at 90%RH. (b) Voltage output tests of the *G.s*-PSII and the proteinase K treated *G.s*-PSII HPEGs under darkness and light at 90%RH.

For the proteinase K treatment, the *G.s* cell suspension was incubated with 5.0 U/mL proteinase K for 2 hours to digest the out-membrane proteins. Then, the digestion reaction was stopped by adding 5 mM proteinase inhibitor-phenylmethylsulfonyl fluoride (PMSF). Then the cell suspension was centrifugated and washed for three times. The proteinase K treated *G.s* cells were injected into a new mineral medium without acetate. Subsequently, the PSII particles were added into the suspension of the proteinase K treated *G.s* cells. Other operations are similar to those of normal *G.s*.


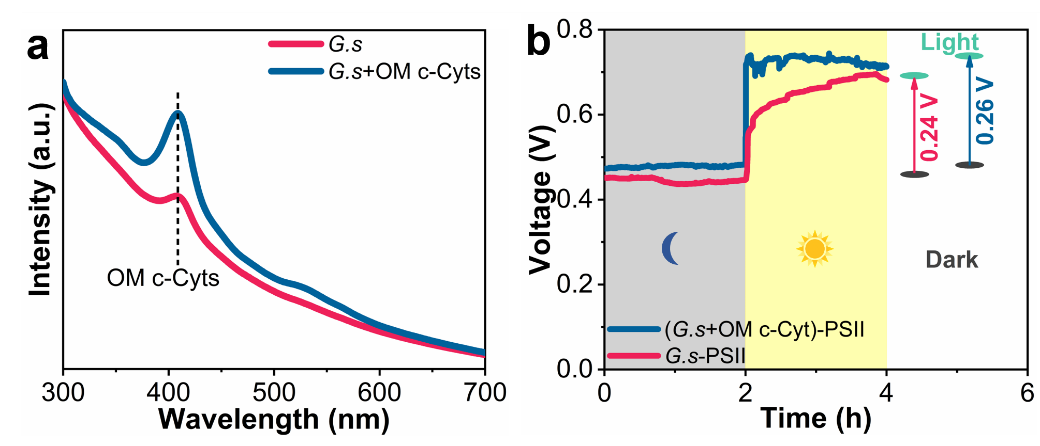


**Figure S18.** The effect of OM c-Cyts addition in *G.s.* on light-response of the *G.s*-PSII HPEG. (a) UV-vis absorption spectra of *G.s* and *G.s* with OM c-Cyts addition. (b) Voltage output tests of the *G.s*-PSII and the (*G.s+*OM c-Cyt)-PSII HPEGs under darkness and light at 90%RH.


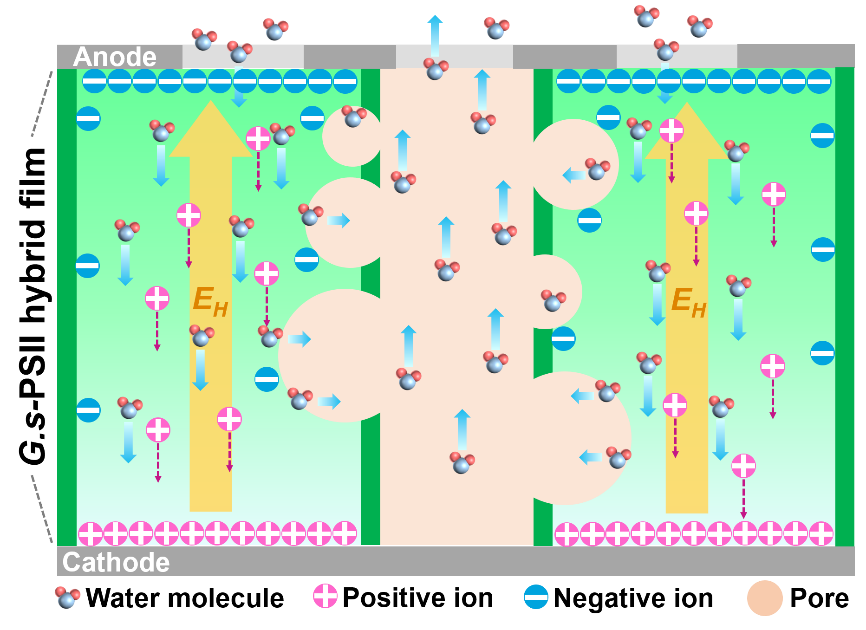


**Figure S19.** The mechanism of hygroelectricity generation for the *G.s*-PSII HPEG.


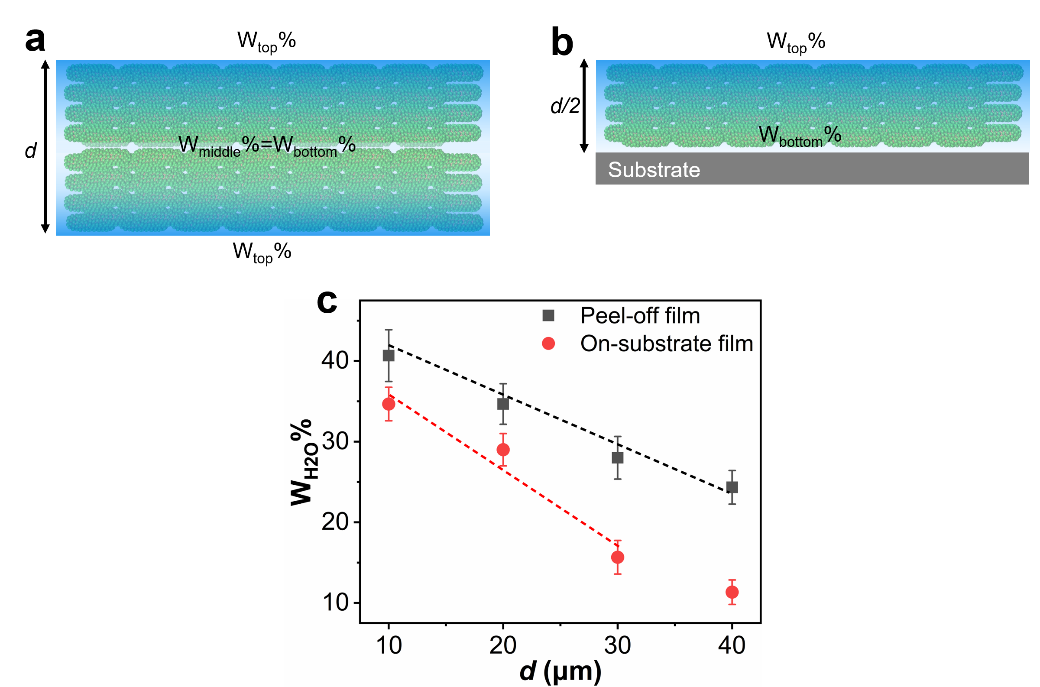


**Figure S20.** Moisture adsorption in peel-off films. The existence of moisture gradient can be further verified by following analysis. If a moisture gradient existed (i.e., the local adsorption would be dependent on its depth from the interface), then exposing both interfaces would yield increased moisture adsorption. Specifically, the average adsorption ratio (W_H2O_%) in a peel-off film (thickness *d*, (a)) is expected to be (W_top_% + W_middle_%)/2, where W_top_% and W_middle_% are the local adsorption ratios at the top and middle interfaces, respectively. For a symmetric diffusion from top and bottom interfaces, W_middle_% is expected to be the same as the bottom adsorption ratio (W_bottom_%) in a half thick (*d*/2) film deposited on substrate (i.e., bottom interface being sealed, (b)). This indicates that (before saturation) the average adsorption ratio in a peel-off film is expected to be similar to that in a half-thick on substrate film, or its thickness dependence (slope) is expected to be half of that in on-substrate films. (c) Measured adsorption ratios in peel-off films at different thicknesses (black dots), compared to those in on-substrate films (red dots). The linear fitting (dashed lines) yielded a slope of -0.55% μm^-1^ in peel-off films, which was close to half the value (-0.95% μm^-1^) in on-substrate films. This result indicated that a moisture gradient existed in the film. Weight changes were measured by electronic balance and quartz crystal microbalance [[1](#_ENREF_1), [2](#_ENREF_2)].


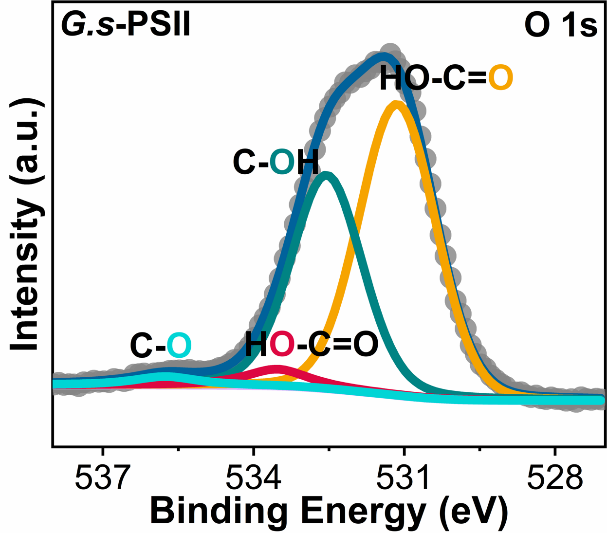


**Figure S21.** XPS oxygen spectra of the *G.s*-PSII hybrid film.


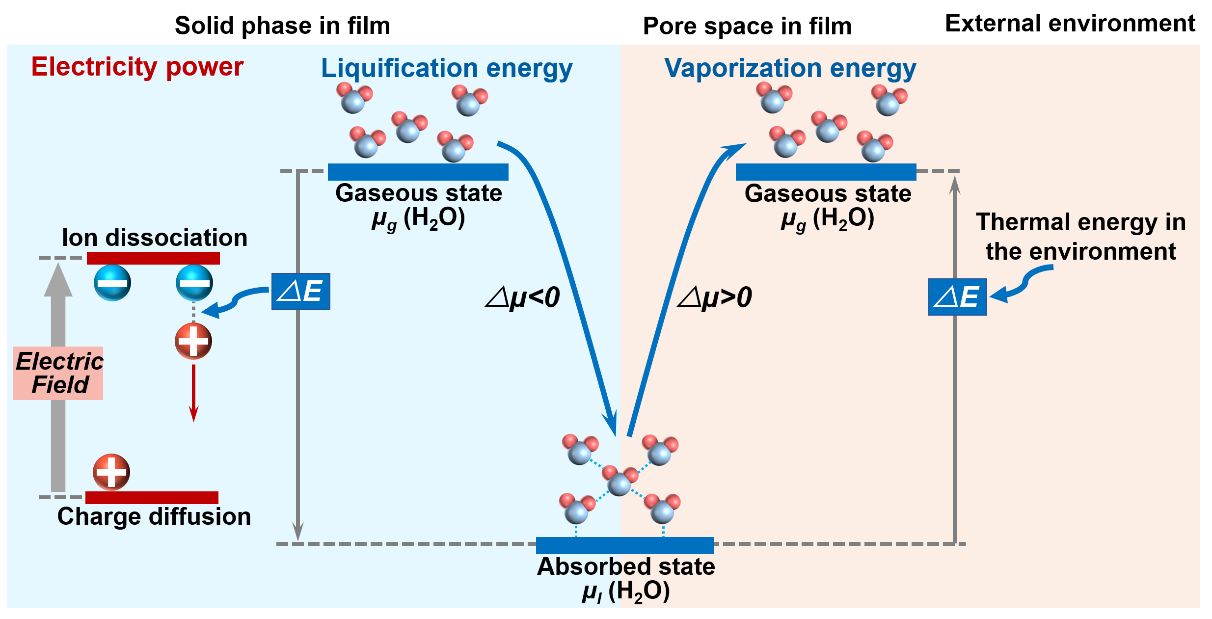


**Figure S22.** Schematic of the energy transformation in hygroelectricity. The internal energy of water in air (*μ_g_*) and in solid phase of film (*μ_l_*) order are: *μ_g_* > *μ_l_*. When the hydrophilic groups in the film contact with moisture, the spontaneous water absorption and liquification release energy (*△*E), which provides energy for ions dissociation. The spontaneous water molecules movement from surface to interior in the film, resulting in charge (ion) diffusion and formation of build-in electric field. The interior water was evaporated by thermal energy from the external environment.


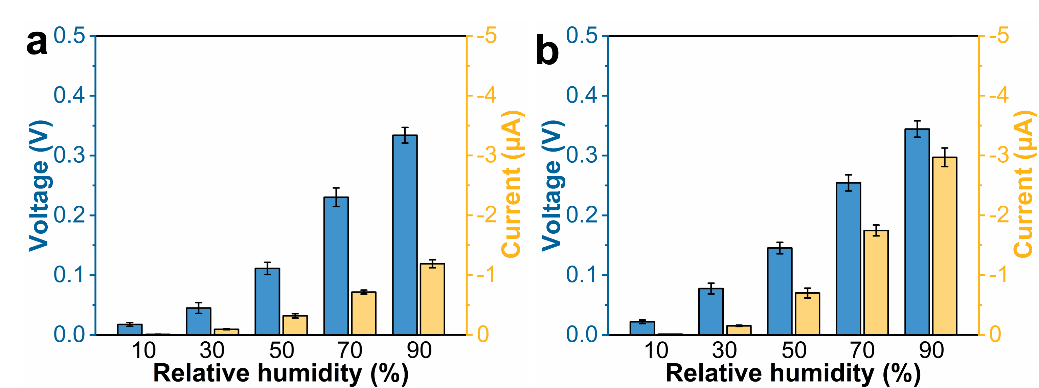


**Figure S23**. The effect of RHs on output voltage and current of the PSII (a) and *G.s* (b) based devices under darkness.


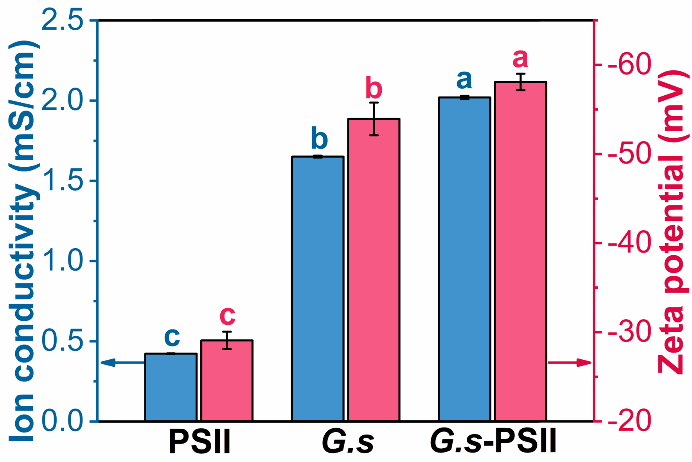


**Figure S24.** Ion conductivity and zeta potential of PSII film, *G.s* film and *G.s*-PSII hybrid film. The different letters represent statistically significant difference (*P* < 0.05).


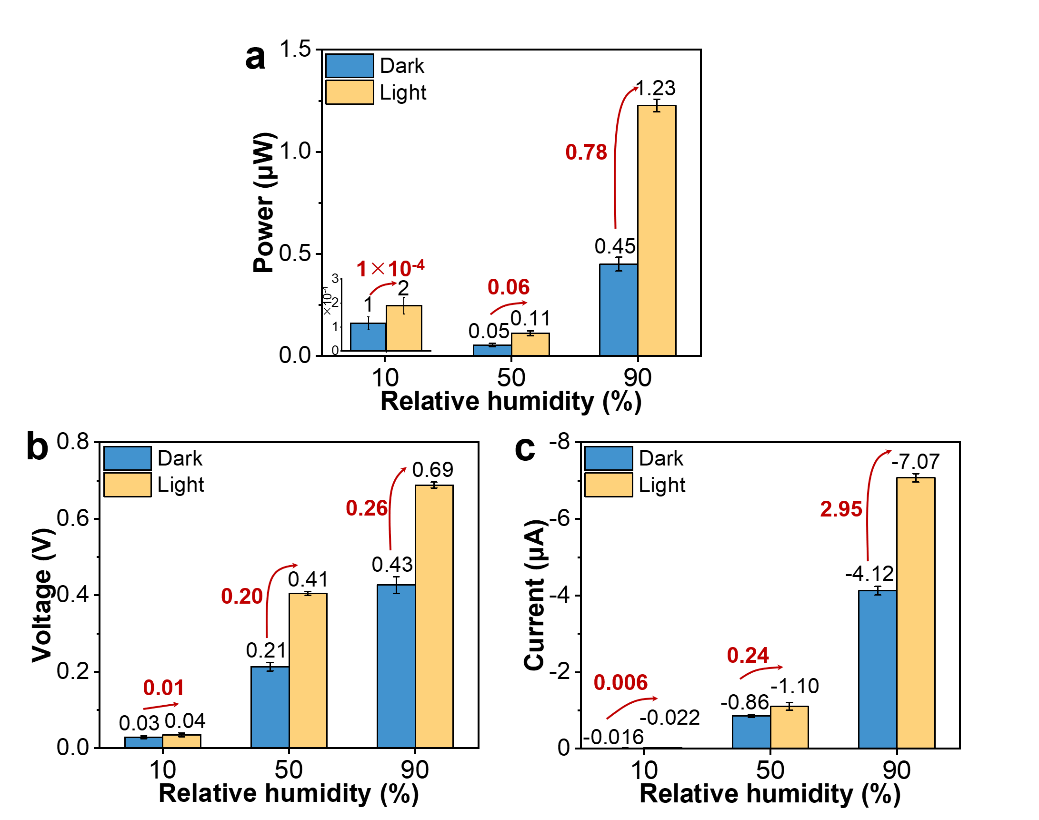


**Figure S25.** Effect of different RHs on electricity generation of the HPEG under darkness and light, respectively. Effect of different RHs on (a) power output, (b) voltage output and (c) current output. The red letter is the increase of the value.

**Table S1.** A comparison of the power density of the device in this work with that of the sustainable hygroelectricity generators in the references.

| **Number** | **Film material** | **Film structure** | **Power density (mW/m^2^)** | **Output type** | **Ref.** |
| --- | --- | --- | --- | --- | --- |
| *Ref.1* | Porous carbon film | Porous structure | 1.0🞨10^-4^ | Continuous | [[3](#_ENREF_3)] |
| *Ref.2* | Cellulose | Porous structure | 2.0🞨10^-2^ | Continuous | [[4](#_ENREF_4)] |
| *Ref.3* | Protein  nanowire | Porous structure | 48.0 | Continuous | [[1](#_ENREF_1)] |
| *Ref.4* | Asymmetric  ionic aerogels | Porous structure | 3.1🞨10^-3^ | Continuous | [[5](#_ENREF_5)] |
| *Ref.5* | Cationic silk nanofibrils | Porous structure | 1.0🞨10^-2^ | Continuous | [[6](#_ENREF_6)] |
| *Ref.6* | Graphite-Cellulose | Porous structure | 2.3 | Continuous | [[7](#_ENREF_7)] |
| *Ref.7* | Cellulose  acetate | Porous structure | 8.0🞨10^-2^ | Continuous | [[8](#_ENREF_8)] |
| *Ref.8* | Whole-cell | Porous structure | 25.0 | Continuous | [[2](#_ENREF_2)] |
| This work | *G.s*-PSII | Porous structure | 1,240 | Continuous |  |

**Table S2.** Fit parameters for the PL decay curves.

| **Treatment** | **A_1_** | **A_1_** | **τ_1_** | **τ_2_** | **τ** |
| --- | --- | --- | --- | --- | --- |
| PSII | 288.42 | 25.00 | 0.54 | 3.46 | 1.58 |
| *G.s* | 368.20 | 28.85 | 0.83 | 3.91 | 1.66 |
| *G.s*-PSII | 647.78 | 55.00 | 0.82 | 4.22 | 1.85 |

The average lifetime of the PL decay is calculated by using following expression:

$$\text{τ = }\frac{\sum\text{(}\text{A}_{\text{n}}{\text{τ}_{\text{n}}}^{\text{2}}\text{)}}{\sum\text{(}\text{A}_{\text{n}}\text{τ}_{\text{n}}\text{)}}$$

where n corresponds to the n^th^ component of a given multi-exponential decay process.[[9](#_ENREF_9)]

**Table S3.** NBAF media for *Geobacter sulfurreducens.*

| **Ingredient** | **1 L** |
| --- | --- |
| Milli-Q H_2_O | 800 mL |
| Fumaric Acid | 4.64 g |
| 100🞨 NB Salts | 10 mL |
| NB Mineral Elixir | 10 mL |
| DL Vitamins | 15 mL |
| CaCl_2_·2H_2_O | 0.04 g |
| MgSO_4_·7H_2_O | 0.1 g |
| NaHCO_3_ | 1.8 g |
| Na_2_CO_3_·H_2_O | 0.5 g |
| 1mM Na_2_SeO_4_ | 1.0 mL |
| Na Acetate·3H_2_O | 2.04 g |
| Complete volume with Milli-Q H_2_O to | 1000 mL |

**Table S4.** 100🞨 NB Salts Solution, NB Mineral Elixir Solution and DL Vitamins Solution of NBAF media of *Geobacter sulfurreducens.*

| **100🞨 NB Salts** | | **DL Vitamins Solution** | | **NB Mineral Elixir Solution** | |
| --- | --- | --- | --- | --- | --- |
| **Ingredient** | **1 L** | **Ingredient** | **1 L** | **Ingredient** | **1 L** |
| Milli-Q H_2_O | 800 mL | Milli-Q H_2_O | 800 mL | Milli-Q H_2_O | 800 mL |
| KH_2_PO_4_·H_2_O | 42 g | Biotin | 0.002 g | NTA | 2.14 g |
| K_2_HPO_4_·2H_2_O | 22 g | Pantothenic Acid | 0.005 g | MnCl_2_·4H_2_O | 0.1 g |
| NH_4_Cl | 20 g | B-12 | 0.0001 g | FeSO_4_·7H_2_O | 0.3 g |
| KCl | 38 g | p-aminobenzoic Acid | 0.005 g | CoCl_2_·6H_2_O | 0.17 g |
|  |  | Vitamin Box |  | ZnSO_4_·7H_2_O | 0.2 g |
|  |  | Thioctic Acid | 0.005 g | CuCl_2_·2H_2_O | 0.03 g |
|  |  | Nicotinic Acid | 0.005 g | AlK(SO_4_)_2_·12H_2_O | 0.005 g |
|  |  | Thiamine | 0.005 g | H_3_BO_3_ | 0.005 g |
|  |  | Riboflavin | 0.005 g | Na_2_MoO_4_·2H_2_O | 0.09 g |
|  |  | Pyridoxine HCl | 0.01 g | NiSO_4_·6H_2_O | 0.11 g |
|  |  | Folic Acid | 0.002 g | Na_2_WO_4_·2H_2_O | 0.02 g |

**Movie S1.** The integrated HPEG directly powered the electronic ink screen under cloudy sky.

**Movie S2.** The integrated HPEG directly powered the electronic ink screen under rainy day.

**Movie S3.** The integrated HPEG directly powered the electronic ink screen under sunlight.

**Supporting References**

[1] X. Liu, H. Gao, J. E. Ward, X. Liu, B. Yin, T. Fu, J. Chen, D. R. Lovley, J. Yao, Power generation from ambient humidity using protein nanowires. *Nature*, vol. 578, no. 7796, pp. 550-554, 2020.

[2] G. Ren, Z. Wang, B. Zhang, X. Liu, J. Ye, Q. Hu, S. Zhou, A facile and sustainable hygroelectric generator using whole-cell *Geobacter sulfurreducens*. *Nano Energy*, vol. 89, no., pp. 106361, 2021.

[3] K. Liu, P. Yang, S. Li, J. Li, T. Ding, G. Xue, Q. Chen, G. Feng, J. Zhou, Induced potential in porous carbon films through water vapor absorption. *Angewandte Chemie International Edition*, vol. 55, no. 28, pp. 8003-8007, 2016.

[4] M. Li, L. Zong, W. Yang, X. Li, J. You, X. Wu, Z. Li, C. Li, Biological nanofibrous generator for electricity harvest from moist air flow. *Advanced Functional Materials*, vol. 29, no. 32, pp. 1901798, 2019.

[5] W. Yang, X. Li, X. Han, W. Zhang, Z. Wang, X. Ma, M. Li, C. Li, Asymmetric ionic aerogel of biologic nanofibrils for harvesting electricity from moisture. *Nano Energy*, vol. 71, no., pp. 104610, 2020.

[6] W. Yang, L. Lv, X. Li, X. Han, M. Li, C. Li, Quaternized silk nanofibrils for electricity generation from moisture and ion rectification. *ACS Nano*, vol. 14, no. 8, pp. 10600-10607, 2020.

[7] K. S. Moreira, D. Lermen, L. P. dos Santos, F. Galembeck, T. A. Burgo, Flexible, low-cost and scalable, nanostructured conductive paper-based, efficient hygroelectric generator. *Energy & Environmental Science*, vol. 14, no. 1, pp. 353-358, 2021.

[8] Q. Lyu, B. Peng, Z. Xie, S. Du, L. Zhang, J. Zhu, Moist-induced electricity generation by electrospun cellulose acetate membranes with optimized porous structures. *ACS Applied Materials & Interfaces*, vol. 12, no. 51, pp. 57373-57381, 2020.

[9] D. R. James, Y.-S. Liu, P. De Mayo, W. R. Ware, Distributions of fluorescence lifetimes: consequences for the photophysics of molecules adsorbed on surfaces. *Chemical Physics Letters*, vol. 120, no. 4-5, pp. 460-465, 1985.
